# Van der Waals Heterostructures for Next‐Generation Spintronics: Multiferroic‐Mediated Magnetoelectric Properties

**DOI:** 10.1002/advs.77210

**Published:** 2026-08-21

**Authors:** Donghyeon Lee, Jiwoong Yang, Inhyeok Oh, Sanghan Lee

**Affiliations:** ^1^ Department of Materials Science and Engineering Gwangju Institute of Science and Technology Jeonnam‐Gwangju Special Metropolitan City Republic of Korea; ^2^ UNIST InnoCORE AI‐Space Solar Initiative Ulsan National Institute of Science and Technology (UNIST) Ulsan Republic of Korea; ^3^ Research Institute for Solar and Sustainable Energies (RISE) Gwangju Institute of Science and Technology Special Metropolitan City Republic of Korea

**Keywords:** magnetoelectric coupling, multiferroic heterostructures, spintronics, van der Waals materials, voltage control of magnetism

## Abstract

The growing demand for energy‐efficient information processing is pushing conventional complementary metal‐oxide‐semiconductor (CMOS) technology toward its fundamental limits, driving the search for alternative material platforms. Among these, magnetic systems are attractive for next‐generation devices because they can store and transmit information via spin transport. However, manipulating magnetization states with electric currents remains intrinsically energy intensive. Multiferroic heterostructures, which combine ferroelectric and ferromagnetic orders, provide a promising route toward low‐power electronics by enabling electric‐field control of magnetic order with 2–3 orders of magnitude lower energy dissipation than current‐driven schemes. In this review, we summarize recent progress in the electrical control of magnetism using artificial multiferroic heterostructures based on conventional oxides and emerging van der Waals (vdW) two‐dimensional (2D) materials, highlighting interfacial coupling mechanisms such as magnetic anisotropy modulation, strain transfer, and the Dzyaloshinskii–Moriya interaction (DMI). Finally, we discuss fabrication strategies for vdW 2D multiferroic heterostructures that are critical for future device integration, including phase‐engineered synthesis, contamination‐free assembly, and passivation schemes for environmental stability.

## Graphical abstract

As CMOS technology approaches fundamental energy limits, new paradigms for low‐power information processing are required. Magnetic systems are attractive for their spin transport, yet current‐induced magnetization control is energy inefficient. Multiferroic heterostructures enable energy‐efficient electric field manipulation of magnetism. This review summarizes the advances in oxide and emerging van der Waals two‐dimensional multiferroic heterostructures, coupling mechanisms, and fabrication routes.

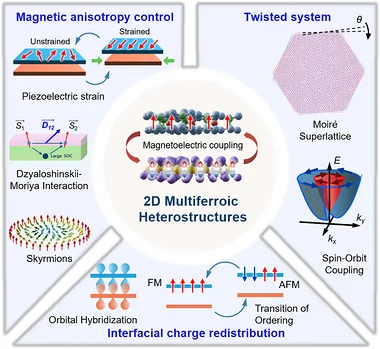

## 1 Introduction

The relentless demand for high‐performance computing has driven the rapid evolution of Si‐based complementary metal‐oxide‐semiconductor (CMOS) technology. Exemplified by the metal‐oxide‐semiconductor field‐effect transistor (MOSFET), this platform has satisfied the ever‐increasing requirements of modern information processing for decades [1, 2, 3]. However, conventional MOSFET technology now faces a fundamental physical limit, often referred to as the Boltzmann tyranny, owing to its switching mechanism based on the thermally activated emission of electrons over an energy barrier [4]. This constraint fixes the subthreshold swing near 60 mV dec^−1^ at room temperature, hindering further supply‐voltage scaling and causing power consumption to grow approximately linearly with clock frequency [5]. Moreover, in traditional von Neumann architectures, data must be continuously shuttled between physically separated memory and processing units, leading to latency and substantial energy dissipation. Taken together, these constraints expose the limitations of charge‐based, volatile switching in conventional CMOS technology and motivate the exploration of beyond‐CMOS device concepts in which information is stored in internal order parameters rather than mobile charge. In this context, ferroic materials have emerged as key beyond‐CMOS candidates. Owing to their switchable spontaneous order, they can retain information without external power. Moreover, their polarization, magnetization, or strain can serve as non‐volatile state variables. The reconfiguration of these order parameters through electric or magnetic fields, rather than energy‐intensive charge currents, enables low‐power switching. Such field‐driven, non‐volatile order facilitates the development of novel architectures that simultaneously alleviate the physical scaling limits of MOSFETs and resolve the fundamental bottlenecks in modern computing [6].

Ferroic materials are generally classified into three types according to their order parameters: ferroelectrics, which exhibit spontaneous polarization that can be switched by an electric field; ferromagnets, which possess magnetization that can be reoriented by a magnetic field; and ferroelastics, which display spontaneous strain that can be reconfigured by mechanical stress. Among these ferroic orders, spintronics based on ferromagnetism exploits the electron spin degree of freedom, in contrast to the conventional charge‐based operation. This approach inherently offers non‐volatile and energy‐efficient functionality, while the robustness of spin dynamics enables fast switching and extremely high endurance [7]. These advantages have driven the successful deployment of spintronics technologies, from hard disk drives (HDDs) to magnetic random‐access memory (MRAM), which is now recognized as a key technology in the International Roadmap for Devices and Systems (IRDS) [8, 9]. Nevertheless, conventional spintronics typically rely on electric currents for magnetization switching. Although spin‐transfer torque MRAM (STT‐MRAM) has demonstrated the commercial viability of spintronics, it inevitably suffers from Joule heating and energy dissipation associated with the high current densities required for reliable switching [10, 11]. This current‐driven nature fundamentally limits further reduction in switching energy.

Multiferroics, which exhibit coexisting magnetic and electric orders in a single phase, are regarded as a promising route to overcome the limitations of current‐induced magnetization switching [12, 13, 14]. The exploration of these coupled phenomena is built upon a rich history of fundamental discoveries. The field originated with the seminal theoretical prediction by Dzyaloshinskii [15], who used thermodynamic symmetry arguments to predict the linear magnetoelectric (ME) effect in antiferromagnetic Cr_2_O_3_. This was rapidly validated by the first experimental observations of magnetization induced by an electric field by Astrov [16] and magnetic‐field‐induced polarization by Folen et al. [17] The pursuit of such materials was later revitalized and formally unified under the concept of multiferroics by Schmid [18]. Depending on the microscopic origin of this coupling, multiferroics are commonly classified into two types. In type‐I multiferroics, the ferroelectric and magnetic orders arise from largely independent ions or mechanisms and therefore coexist with a weak coupling. In type‐II multiferroics, by contrast, ferroelectricity is induced directly by a particular magnetic order, producing strong intrinsic coupling; for example, antisymmetric exchange interactions generate electric polarization through spiral spin configurations [19]. This coexistence gives rise to ME coupling, which enables not only the control of electric polarization by a magnetic field but also the manipulation of magnetization by an electric field [20]. Exploiting such ME coupling allows voltage‐driven magnetization switching, potentially enhancing switching speed and reducing energy consumption by more than an order of magnitude compared with current‐driven schemes [21, 22]. However, realizing robust multiferroicity in single‐phase materials remains a formidable challenge due to a fundamental chemical incompatibility that ferroelectricity typically favors empty *d* orbitals, whereas magnetism requires partially filled *d* orbitals [23, 24]. BiFeO_3_ (BFO) is the most prominent example of a room‐temperature single‐phase multiferroic, yet its practical application is limited by its weak net ferromagnetism arising from a spin‐cycloid structure induced by the Dzyaloshinskii–Moriya interaction (DMI), which in turn suppresses the effective ME coupling [25, 26]. As a result, single‐phase multiferroics alone are insufficient to meet the demands of low‐power, high‐reliability spintronics.

As research subsequently progressed to overcome the scarcity, intrinsic limitations, and inherently weak ME coupling coefficient of single‐phase bulk materials, significant milestones were achieved in interfacial ME coupling. Foundational theoretical models based on density functional theory by Duan et al. [27] demonstrated that modifying the interfacial bonding at a ferromagnet/ferroelectric junction could significantly alter the surface magnetization. This was complemented by pivotal experimental demonstrations; for instance, Borisov et al. [28] successfully observed the tuning of exchange bias via external electric fields, while Laukhin et al. [29] reported the non‐volatile macroscopic control of giant magnetoresistance using an adjacent ferroelectric layer. Building upon this profound historical context and theoretical foundation, artificial multiferroic heterostructures composed of distinct ferroelectric and ferromagnetic layers have emerged as a powerful alternative to single‐phase systems. By assigning the ferroelectric and ferromagnetic functionalities to chemically distinct, spatially separated layers, this architecture circumvents the contradictory *d*‐orbital occupancy requirements that preclude the coexistence of the two orders in a single phase; the ME response is instead generated across the interface through the coupling of spin, charge, orbital, and lattice degrees of freedom. This strategy dramatically broadens the accessible material space and enables rich interfacial phenomena, which are absent in the individual bulk constituents [30, 31]. Furthermore, by carefully combining different ferroic components, strong ME responses can be engineered at ambient conditions, thereby mitigating the constraints of single‐phase systems [32]. To date, most experimental demonstrations of such artificial multiferroics have relied on oxide‐based heterostructures, which provide excellent structural compatibility and robust ferroic orders. However, they suffer from the lack of interface sharpness, hard defect control, and scaling issues. These challenges motivate the search for alternative platforms in which interfacial coupling can be engineered with atomic precision and new regimes of multiferroicity can be explored.

In this context, van der Waals (vdW) two‐dimensional (2D) materials have rapidly emerged as an intriguing platform for multiferroic heterostructures [33]. Their unique electronic structures and strong quantum confinement effect provide opportunities to significantly enhance ME coupling and to realize emergent quantum phases that are absent in conventional oxide systems [33]. At the same time, the weak interlayer bonding of vdW materials allows the preservation of the intrinsic properties of the individual layers, while offering the possibility of tailoring interfacial magnetic and electronic effects [34]. Despite these advantages, single‐phase 2D multiferroics discovered so far often exhibit weak coupling between the order parameters and suffer from thermal instability at elevated temperatures, which severely limit their practical applications [35, 36, 37, 38, 39]. Consequently, constructing ferromagnetic/ferroelectric vdW heterostructures has emerged as a promising route to achieve robust and electrically controlled multiferroicity. Although this spatial decoupling is the defining strategy of all artificial multiferroic heterostructures, vdW systems realize it in a near‐ideal form. The atomically sharp, dangling‐bond‐free vdW interface keeps the two ferroic layers electronically pristine and preserves their intrinsic order, yet the sub‐nanometer interlayer gap is small enough to sustain strong short‐range ME coupling. Specifically, the out‐of‐plane spontaneous polarization of the ferroelectric layer imposes an asymmetric electrostatic potential that drives substantial interfacial charge transfer and band realignment at the adjacent magnetic layer [40]. Reversing this polarization further modifies the interlayer orbital hybridization and the local coordination environment, thereby modulating the interfacial magnetic exchange pathways and anisotropy [41, 42]. In contrast to covalently bonded oxide interfaces, where interdiffusion, dead‐layer formation, and lattice clamping degrade this decoupling, vdW materials are particularly useful for such heterostructures since they are free from lattice mismatch constraints and can be assembled into designated interfaces with controllable stacking sequences, twist angles, and interlayer separations [43, 44]. This provides additional degrees of freedom for engineering 2D multiferroicity and optimizing device performance over the conventional epitaxial systems.

Motivated by these developments, this review examines the multiferroic properties and ME responses of both conventional oxide‐based and emerging 2D material‐based heterostructures in the context of future spintronics applications. Before surveying specific material systems, we briefly summarize the phenomenology of ME coupling that underpins the discussion throughout this review. The linear ME effect introduced above is conventionally expressed as the lowest‐order cross coupling between electric and magnetic fields in the free energy expansion [45]:

(1) $$F\left(E,H\right)=F_{0}-\alpha_{\mathrm{ij}}E_{i}H_{j}-\frac{1}{2}\beta_{\mathrm{ijk}}E_{i}H_{j}H_{k}$$

where α_ij_ is the linear ME tensor. Differentiation of Equation (1) with respect to the applied fields yields the direct and converse ME effects,

(2) $$\Delta P_{i}=\alpha_{\mathrm{ij}}H_{j,}\mu \Delta M_{i}=\alpha_{\mathrm{ji}}E_{j}$$

in which an applied magnetic field induces a change in electric polarization and, conversely, an applied electric field induces a change in magnetization; the converse effect constitutes the operating principle of voltage control of magnetism on which this review centers [46]. The term “multiferroic,” denoting the coexistence of two or more ferroic orders in a single phase, was formalized by Schmid [18]. In composite heterostructures, where the two ferroic orders reside in adjacent layers, the dominant coupling channel is often strain‐mediated and can be expressed by the product relation ∂M/∂E = (∂M/∂ε)·(∂ε/∂E): converse piezoelectric strain generated in the ferroelectric layer is transferred across the interface and converted into a magnetic response through magnetostriction and inverse magnetostriction, also known as the Villari effect [24]. Experimentally, this coupling is frequently reported as the ME voltage coefficient α_E_ = dE/dH (frequently denoted 𝞪_ME_, in V cm^−1^ Oe^−1^), which is distinct from but related to the thermodynamic tensor 𝞪_ij_ and provides a practical metric for comparing the systems discussed in Sections 2 and 3.

We first outline the key mechanisms of ME coupling in conventional oxide‐based heterostructures (Section 2) and vdW 2D heterostructures (Section 3), followed by their electric and magnetic characteristics. We then highlight the fabrication strategies for high‐quality vdW 2D multiferroic heterostructures (Section 4) that are crucial for device integration, including phase‐engineered synthesis, contamination‐free assembly, and passivation schemes. Finally, we conclude with a summary and outlook on design principles for beyond‐classical multiferroics and their prospects for low‐power information technologies.

## 2 Magnetoelectric Coupling and Voltage Control of Magnetism in Conventional Oxide and Emerging Ferroelectric Heterostructures

Over the past several decades, oxide‐based multiferroic heterostructures have served as a primary platform for exploring electric field control of magnetism. A wide range of oxide ferroic materials has been investigated, including Pb(Zr_1‐x_,Ti_x_)O_3_ (PZT), BaTiO_3_ (BTO), BFO, HfO_2_, La_1‐x_Sr_x_MnO_3_ (LSMO), CoFe_2_O_4_ (CFO), and Pb(Mg_1/3_Nb_2/3_)O_3_‐PbTiO_3_ (PMN‐PT) [47, 48, 49, 50, 51, 52, 53]. In essence, such a heterostructure couples two functional constituents, a ferroelectric layer and a ferromagnetic layer, across a shared interface, where electrical switching of the former modulates the magnetic state of the latter. These systems benefit from well‐established thin film deposition techniques, such as pulsed laser deposition (PLD) and sputtering, which provide a practical route toward device integration [54]. In oxide heterostructures, direct chemical bonding across the interface ensures that modulations of spin, charge, orbital states, and lattice strain in one layer strongly affect the adjacent layer [55, 56]. The ferroelectric constituents derive their polarization from fundamentally different microscopic mechanisms, which in turn influence their integration routes and ME coupling behavior. Conventional perovskite ferroelectrics such as BTO and PZT are displacive ferroelectrics, where polarization arises from the off‐center displacement of the B‐site cation enabled by B(d)–O(p) hybridization; because this mechanism generally favors a formally *d* ^0^ configuration, it is one important microscopic origin of the scarcity of single‐phase magnetic ferroelectrics [57]. BFO avoids this *d* ^0^ constraint through the stereochemically active Bi 6*s* ^2^ lone pair on the A site, which drives the polar distortion independently of the magnetic Fe sublattice [58]. Ferroelectricity in HfO_2_‐based films instead originates from the stabilization of a metastable polar orthorhombic Pca2_1_ phase by doping, strain, finite‐size effects, and surface/interface energy contributions at nanoscale thicknesses [59], whereas wurtzite Sc‐substituted AlN switches its polarization along the polar c‐axis, with Sc alloying flattening the energy landscape and reducing the coercive barrier [60].

The ferromagnetic constituents, by contrast, embody the complementary electronic requirement for magnetism. Perovskite manganites such as LSMO are ferromagnetic metals whose magnetic order arises from double exchange between mixed‐valence Mn^3^^+^/Mn^4^^+^ ions mediated by O 2*p* states, yielding a high degree of spin polarization that is sensitive to interfacial charge and orbital reconstruction. Spinel ferrites such as CFO are insulating ferrimagnets with large magnetostriction, making them well suited for coupling mediated by strain. In many heterostructures aimed at device applications, metallic ferromagnets such as Co, CoFeB, and Heusler alloys instead serve as the active magnetic layer, whose anisotropy and coercivity can be tuned through interfacial charge accumulation and strain transfer. In all of these materials, the magnetic order originates from partially filled 3*d* states and the associated exchange interactions. This electronic configuration is precisely what conflicts with the *d* ^0^ preference of displacive ferroelectricity. This complementarity is the very basis of the heterostructure strategy, in which each constituent independently fulfills one ferroic order while the ME response emerges at their interface.

Building on this framework, we now examine how interfacial ME coupling is exploited and enhanced in representative oxide and ScAlN‐based heterostructures. These studies show that even small interfacial variations, such as defects, strain, atomic termination, and stacking sequence, can strongly influence the magnetic response, underscoring the need for platforms in which such factors can be precisely controlled or eliminated.

Heterostructures of ferroelectric and ferromagnetic materials have been extensively studied because electric‐field‐induced changes in the ferroelectric layer can switch the correlated spins in the ferromagnetic layer [61, 62, 63, 64, 65, 66, 67]. This voltage control of magnetism (VCM) offers a promising route for enhancing energy efficiency by manipulating magnetic states using leakage‐free electric fields rather than charge currents. There are some representative strategies to exploit ferroelectric switching to modulate the magnetic properties of an adjacent ferromagnetic layer in oxide multiferroic heterostructures. The first is an electrochemical route, in which ion migration within the ferroelectric alters the chemical composition of the magnetic layer. Oxygen ion migration under an electric field is inherent to many oxides, but its integration into functional devices is hindered by the challenges of slow kinetics, uncontrolled migration, and poor stability [68]. Recent studies have shown that ferroelectric‐mediated ion migration can overcome these limitations, offering enhanced controllability and fast switching suitable for device applications [62, 69, 70].

The second is an electronic, charge‐mediated route, in which the ferroelectric polarization together with the atomic stacking sequence at the interface dictates charge accumulation, thereby modifying the interfacial exchange interactions and orbital occupation that govern the magnetization and magnetic anisotropy of the adjacent layer [64, 66]. In both cases, the strength and reversibility of the ME coupling are ultimately governed by the atomic‐scale structure and defect configuration at the interface, making interface quality the decisive factor for reproducible VCM.

**FIGURE 1:**
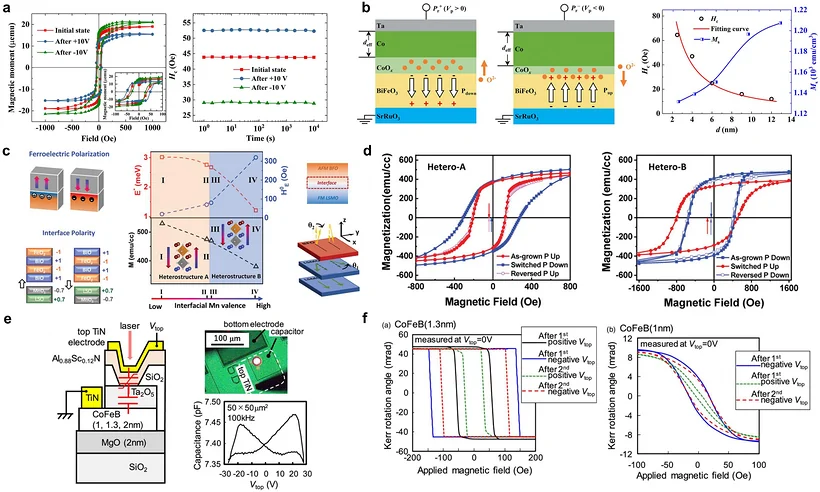
Reversible magneto‐ionic control of magnetism driven by ferroelectric polarization switching in Ta/Co/BiFeO_3_ heterostructures. (a) Left: Magnetic hysteresis loops measured via the vibrating sample magnetometer (VSM) for the initial and polarized states. The inset is the expanded view of the hysteresis loop. Right: Coercive field (*H*_C_) retention behavior over 10^4^ s, measured using the magneto‐optic Kerr effect (MOKE) technique. (b) Left: Illustration of oxygen ion migration and the associated structural evolution of Co and CoO_x_ layer. Right: Effective CoO_x_ thickness dependent magnetic property, specifically *H*_C_ and saturation magnetization (*M*_s_). Reproduced with permission [62]. Copyright 2022, The Chinese Ceramic Society. (c) Left: Illustration of screening effect for ferroelectric polarization and interfacial polar discontinuity within two BiFeO_3_/La_0.7_Sr_0.3_MnO_3_ heterostructures. Heterostructure A features a BiO/MnO_2_ atomic sequence at the interface, while Heterostructure B consists of a FeO_2_/(La,Sr)O interface configuration. Middle: Collected magnetic data at 10 K, including magnetization (*M*), thermal stability of exchange bias (*E* ^*^), and the magnitude of exchange bias (*H*_E_) evaluated across the four distinct configurations (I–IV). The horizontal axis represents the interfacial Mn valence state corresponding to each configuration. Right: Proposed spin reconstruction model at the ferromagnetic/antiferromagnetic interface involving La_0.7_Sr_0.3_MnO_3_ and BiFeO_3_. (d) Magnetic field‐dependent magnetization curves for Heterostructure A and Heterostructure B, measured at 10 K in their initial as‐grown condition. Reproduced with permission [64]. Copyright 2019, John Wiley and Sons. (e) Cross‐sectional diagram and optical top view of the Ta_2_O_5_/Al_0.88_Sc_0.12_N ferroelectric capacitor with a Co_0.2_Fe_0.6_B_0.2_ bottom electrode. Despite the presence of the Ta_2_O_5_ layer, *C*–*V* characteristics display a typical ferroelectric butterfly curve. (f) Magnetic hysteresis loops obtained via Kerr rotation measurements for 1.3 nm and 1 nm thick Co_0.2_Fe_0.6_B_0.2_ layers following the application of positive or negative voltages. Reproduced under terms of the CC‐BY license [66]. Copyright 2025, Yan Wu et al., published by The Japan Society of Applied Physics.

Ferroelectric polarization reversal enables high‐speed magneto‐ionic control, effectively overcoming the kinetic bottlenecks of ion diffusion that typically restrict the switching time of conventional devices to the order of seconds. Chen et al. demonstrated room‐temperature VCM effects in Ta/Co/BFO/SrRuO_3_ heterostructures, where clear changes in magnetic hysteresis loops were induced by 1 ms voltage pulses together with excellent non‐volatile retention (Figure 1a) [62]. Specifically, switching the BFO to the down polarization state with a +10 V bias increased the magnetic coercive field (*H*_C_) and reduced the magnetic moment, whereas the opposite trend was observed under –10 V (up polarization state). These magnetic states remained stable for 10^4^ s without significant degradation, exhibiting superior stability compared to conventional magneto‐ionic devices without ferroelectric layers [68]. This reversible magnetic modulation is attributed to oxygen ion migration and interfacial oxidation coupled with the ferroelectric polarization direction (Figure 1b). In the down polarization state, oxygen ions migrate from the BFO to the Co layer driven by the external electric field and the repulsive force of interfacial polarization charges, forming an antiferromagnetic CoO_x_ layer and consequently reducing the effective thickness of the ferromagnetic Co layer. When the polarization is switched upward, oxygen ions migrate back into BFO and CoO_x_ is reduced to metallic Co. Therefore, the resulting changes in coercivity and magnetic moment are primarily governed by variations in the functional thickness of the ferromagnetic layer. This finding highlights the critical role of the interfacial CoO_x_ layer in mediating ME coupling within the Co/BFO heterostructure.

Yi et al. demonstrated that the atomic stacking sequence at the BFO/LSMO interface is a critical factor to determine the efficiency of ME coupling [64]. Magnetometry measurements revealed reversible changes in magnetization and exchange bias when the BFO polarization was switched from downward to upward; however, the magnitude of this modulation was highly dependent on the interface configuration. Figure 1c shows how atomic‐level interface engineering influences the resulting ME coupling. The BFO/LSMO interface is classified into Heterostructure A, characterized by the junction of the BiO plane of BFO and the MnO_2_ plane of LSMO, and Heterostructure B, where the FeO_2_ and (La,Sr)O planes meet. These configurations lead to as‐grown upward and downward polarizations, respectively. In Heterostructure A, polarization switching promotes electron accumulation at the LSMO interface, thereby inducing high magnetization. In contrast, polarization switching in Heterostructure B leads to hole accumulation and a subsequent strengthening of the antiferromagnetic superexchange interaction. Consequently, Heterostructure B exhibits lower magnetization while simultaneously enhancing the competitive antiferromagnetic superexchange interaction from the BFO layer. These magnetic responses are further evidenced by the hysteresis loops in Figure 1d. Although Heterostructure A possesses a higher magnetization magnitude, Heterostructure B offers a larger magnitude of modulation and increased exchange bias field depending on the polarization state.

ScAlN‐based ferroelectrics provide another CMOS‐compatible route to VCM. Wu et al. recently demonstrated non‐volatile control of *H*_C_ in a CoFeB magnetic layer utilizing the CMOS‐compatible ferroelectric Al_0.88_Sc_0.12_N (ASN) [66]. They fabricated a Ta/CoFeB/MgO stack capped with an ASN ferroelectric layer (Figure 1e) and switched the ASN polarization by applying voltage pulses to the top electrode. The optical micrograph in Figure 1e illustrates the magnetic characterization of the fabricated device using the laser‐based magneto‐optic Kerr effect (MOKE) microscopy system. The butterfly‐shaped capacitance–voltage (*C*–*V*) curve confirms that the device retains its ferroelectric properties even with the incorporation of the Ta_2_O_5_ passivation layer. Figure 1f shows the perpendicular magnetic anisotropy characteristics measured at zero bias after poling. For a 1.3 nm thick CoFeB layer, applying a positive voltage (+20 V) that aligns the polarization downward reduces *H*_C_ to approximately 57 Oe. In contrast, a negative voltage (–20 V) that switches the polarization upward increases *H*_C_ to 141 Oe, which corresponds to a 2.5‐fold enhancement. This modulation is reproducible under subsequent voltage cycles and is attributed to changes in magnetic anisotropy energy (MAE) driven by the variation of electron occupation at the CoFeB layer by electric field control. Notably, the polarization‐induced modulation of *H*_C_ significantly diminishes in the 1 nm CoFeB layer. This is attributed to the depletion of interfacial electrons in the CoFeB layer driven by the downward polarization of the ASN layer. These results provide an experimental demonstration that the *H*_C_ of a magnetic layer can be significantly controlled with non‐volatility by pure voltage through ASN ferroelectric polarization, without the need for charge currents.

Collectively, these studies demonstrate that switching of the ferroelectric polarization can be efficiently transmitted to the adjacent magnetic layer through interfacial charge accumulation, orbital reconstruction, and magneto‐ionic composition changes, establishing this interfacial coupling as the core basis of VCM and a powerful route to enhance the ME effect [62, 64, 66, 67]. The integration of emerging ferroelectrics such as HfO_2_ and ScAlN has further broadened this design space by adding CMOS‐compatible, non‐volatile routes that operate at room temperature. At the same time, the strong sensitivity of these responses to interfacial defects, strain fields, and atomic termination remains a fundamental obstacle to realizing reproducible and scalable oxide devices, which motivates the transition toward alternative platforms such as vdW 2D heterostructures, where interfaces can be engineered with atomic precision and free from the lattice constraints of epitaxial oxides.

## 3 VdW 2D‐Based Multiferroic Heterostructures

Building on the oxide‐based platforms discussed in Section 2, vdW 2D ferroics have recently emerged as a complementary route to realize multiferroicity. Their atomically sharp, dangling bond‐free interfaces and intrinsic scalability down to the monolayer limit overcome the issues of conventional bulk heterostructures, such as interface roughness, defect controllability, and scaling. As a result, vdW 2D systems are attractive platforms for exploring ME coupling at the ultimate thickness limit for next‐generation multiferroics. Several strategies have been proposed to realize stable 2D multiferroicity. One route is to enhance the density of unpaired spins and break the inversion symmetry by introducing transition metal dopants (e.g., Co, Cr, Ni) or rare‐earth elements, as well as defect engineering and intercalation. A second route is to manipulate ferroic order by controlling the carrier concentration via ionic gating or electrostatic doping. Finally, combining room‐temperature operating ferromagnets and ferroelectrics into vdW heterostructures enables interfacial coupling effects such as exchange bias, charge transfer, and orbital reconstruction, which will be the focus of this section. Indeed, recent demonstrations of intrinsic vdW 2D ferroelectrics [39, 71, 72, 73, 74] and ferromagnets [75, 76, 77] have sparked the development of vdW 2D multiferroic heterostructures.

Historically, the realization of long‐range ferroic order in 2D systems was considered elusive. According to the Mermin–Wagner theorem (1966), thermal fluctuations in isotropic 2D Heisenberg systems should preclude spontaneous long‐range magnetic order at finite temperatures [78]. Consequently, early work on 2D ferroics focused on theoretical predictions or quasi‐2D films clamped to the substrates. However, this paradigm shifted dramatically in 2017 with the experimental demonstration of intrinsic 2D ferromagnetism in monolayer CrI_3_ [77] and bilayer Cr_2_Ge_2_Te_6_ [76]. In these materials, magnetic anisotropy opens a gap in the spin‐wave excitation spectrum, stabilizing magnetic order against thermal fluctuations. Around the same period, robust room‐temperature 2D ferroelectricity was also experimentally confirmed in atomic layers of SnTe [74] and In_2_Se_3_ [72], overcoming the depolarization field constraints. The origins of 2D ferroelectricity are currently understood by ionic displacement by phase transition (e.g., SnTe [74], CuInP_2_S_6_ [79]) and relative equilibrium position of intralayer atoms (e.g., In_2_Se_3_ [72, 80]). Another recently emerging mechanism responsible for 2D ferroelectricity is sliding, which has the potential to artificially tune out‐of‐plane polarization of 2D materials by controlling the interlayer slipping, experimentally demonstrated in bilayer boron nitride [73, 81, 82] and 3R‐MoS_2_ [83, 84]. Although the mechanism of sliding ferroelectricity is not clearly understood, it is believed that net interlayer charge transfer due to the inequality of vdW bilayer mainly attributes the ferroelectricity [85]. These breakthroughs triggered an explosion of research into vdW ferroics and shifted the focus from the existence of 2D ferroic order to the engineering and device applications of vdW ferroics.

Despite the existing challenges of vdW 2D ferroelectrics and ferromagnets, such as thermal fluctuations of 2D ferromagnets [86] and the depolarization field of 2D ferroelectrics [87], constructing heterostructures of them provides several advantages via interfacial interactions as previously mentioned above [33, 88]. In this section, we focus on the emerging multiferroic heterostructures that couple vdW 2D ferromagnets with ferroelectric layers, both 3D and vdW 2D, and discuss the proposed mechanisms of ME properties driven by ferroelectric polarization control. We also briefly introduce twist‐engineered vdW 2D heterostructures as a new avenue for designing interfacial multiferroicity.

### 3.1 VdW 2D Ferromagnet/3D Ferroelectric Multiferroic Heterostructures

**FIGURE 2:**
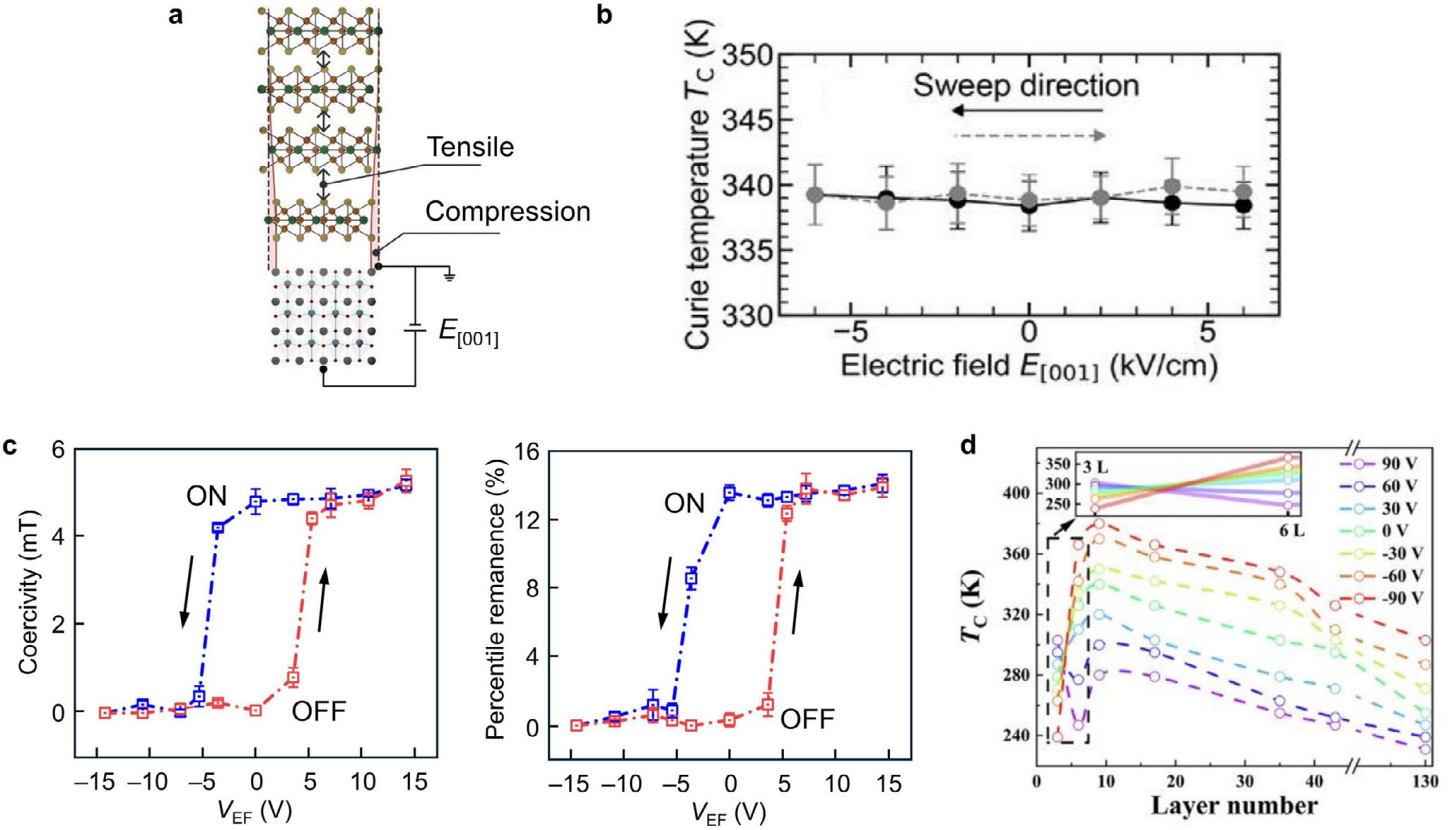
(a) Strain profile generated by the electric field in Fe_3_GaTe_2_/PMN‐PT heterostructure. (b) Unchanged Curie temperature (*T*_C_) with electric field of *E*_[001]_ = 6 kV cm^−1^, indicating the absence of ferromagnetic exchange interactions. Reproduced under terms of the CC‐BY license [89]. Copyright 2025, Iimori et al., published by John Wiley and Sons. (c) Magnetic coercivity (left) and percentile remanence (right) of P(VDF‐TrFE)/bilayer Cr_2_Ge_2_Te_6_ heterostructure as a function of *V*_EF_, the effective voltage dropping only across the heterostructure. Reproduced with permission [44]. Copyright 2023, Springer Nature. (d) Layer number‐dependent *T*_C_ in P(VDF‐TrFE)/Fe_3_GaTe_2_ heterostructure where monolayer corresponds to ∼0.8 nm thick. Reproduced with permission [91]. Copyright 2025, Springer Nature.

The piezoelectric effect of ferroelectrics enables strain‐mediated control of magnetism solely by electric fields, avoiding current dissipation and offering a promising strategy for non‐volatile memory technologies as discussed in Section 2. In addition, the anisotropic piezoelectric strain and the resulting strain gradients offer an additional symmetry breaking in vdW ferromagnets, potentially leading to novel magnetic phenomena [89]. A representative example is the Fe_3_GaTe_2_/PMN‐PT heterostructure reported by Iimori et al., where the magnetic domain structure of Fe_3_GaTe_2_ is controlled by the inverse piezoelectric effect of PMN‐PT at room temperature [89]. The in‐plane strain generated by PMN‐PT leads to an expansion along the c‐axis within the vdW gap, enhancing the perpendicular magnetic anisotropy (Figure 2a) [90]. Additionally, the strain gradients induced by the strain relaxation would break the symmetry along the out‐of‐plane direction and generate the DMI, without ferromagnetic exchange interaction in Fe_3_GaTe_2_ confirmed by nearly unchanged Curie temperature under the electric field (Figure 2b). These results highlight the strong magnetoelastic coupling in vdW ferromagnets and underscore their potential for low‐power spintronics.

Besides the effect of strain‐induced symmetry breaking on the magnetic properties, the interfacial ferroelectric‐ferromagnetic coupling effects also play a significant role by competing with interlayer and intralayer magnetic exchange interactions driven by the electric field. For instance, Liang et al. demonstrated the non‐volatile and direct voltage switching of magnetization of P(VDF‐TrFE)/Cr_2_Ge_2_Te_6_ heterostructure with controllable *H*_C_ and remanent magnetization [44]. The non‐volatile magnetic modulation is possible when ferromagnetic Cr_2_Ge_2_Te_6_ is bilayer (Figure 2c), trilayer, and four‐layer, but vanished in an eight‐layer. This highlights the role of the interfacial multiferroic effect on the magnetic properties. By flipping the ferroelectric polarization direction, the interfacial hybridization alters the crystal field of 2D magnets, leading to interfacial magnetic switching. Cai et al. also investigated the layer number dependence of the ferromagnet layer, using a P(VDF‐TrFE)/Fe_3_GaTe_2_ heterostructure [91]. For trilayer Fe_3_GaTe_2_, switching the polarization from negative to positive results in an increase in the Curie temperature, while thicker configurations show an opposite trend, as shown in Figure 2d. Several mechanisms, including exchange bias, H ion hybridization, and interlayer spacing, would collectively modulate the carrier concentration of ferromagnetic Fe_3_GaTe_2_, leading to layer‐dependent magnetic responses of the device. These studies demonstrate that 2D ferromagnet/3D ferroelectric heterostructures provide a versatile platform in which strain, charge, and orbital reconstruction at the interface can be harnessed to engineer VCM near room temperature.

### 3.2 VdW 2D Ferromagnet/vdW 2D Ferroelectric Multiferroic Heterostructures

As discussed above, constructing heterostructures of vdW 2D ferromagnets and vdW 2D ferroelectrics offers a promising route to realize 2D multiferroics and ME coupling effects. In such systems, the layers are held together by vdW interactions rather than covalent bonding, which minimizes the interfacial defects and dangling bonds. This raises a fundamental question of whether the weakly bonded vdW interlayer can induce strong ME coupling effects. Here, we review several mechanisms by which vdW ferromagnet/ferroelectric heterostructures can nevertheless exhibit substantial ME effects, including the strain‐mediated anisotropy changes and interfacial orbital hybridizations. Next, we discuss the emergent textures such as skyrmions induced by various mechanisms including orbital hybridizations, DMI, and electronic reconstruction at the interface.

#### 3.2.1 Mechanisms of Magnetoelectric Coupling

**FIGURE 3:**
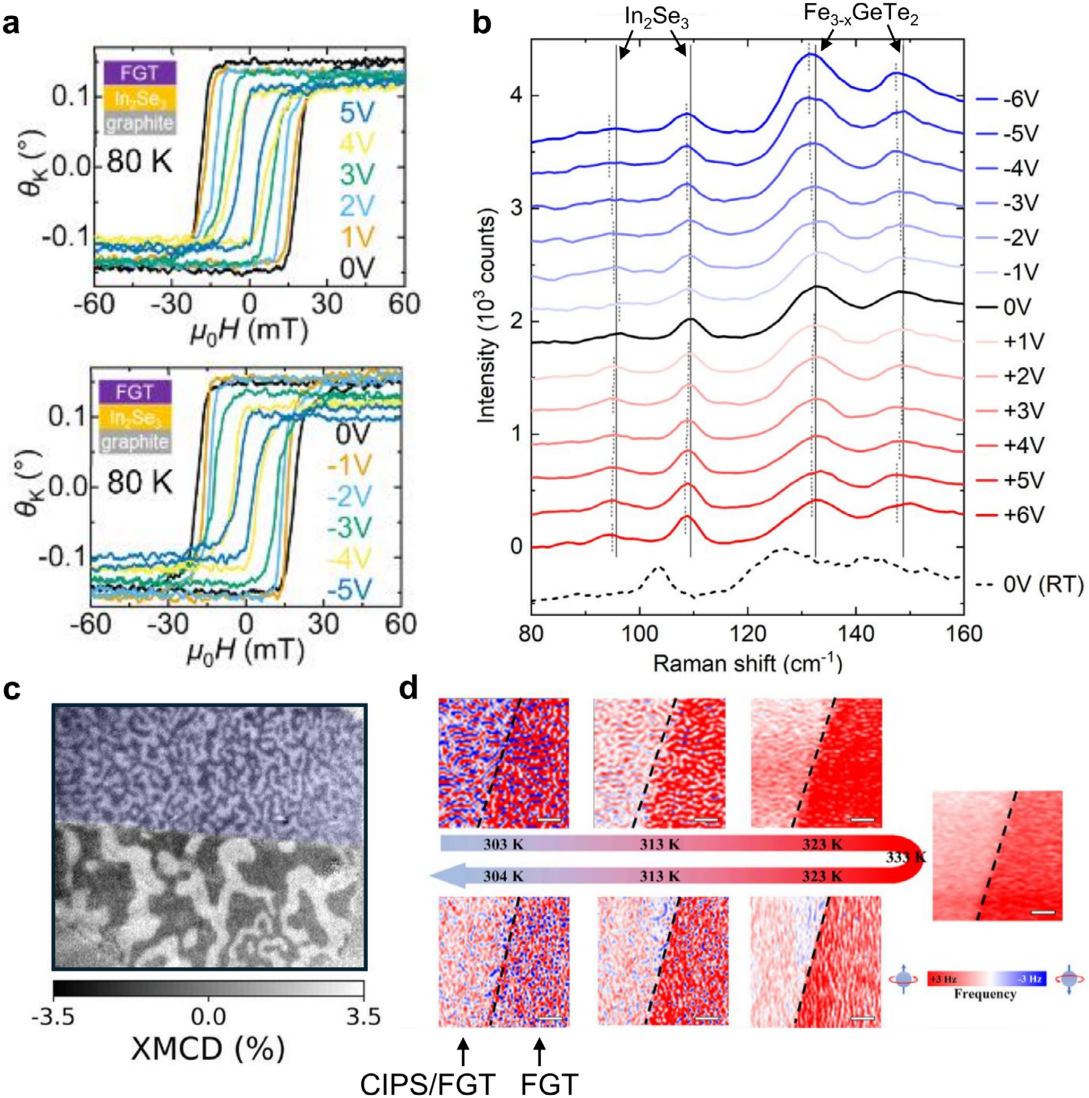
(a) The magnetic hysteresis loops of Fe_3‐x_GeTe_2_/In_2_Se_3_ heterostructure at 80 K for positive (top) and negative (bottom) gate bias measured by magneto‐optic Kerr effect (MOKE) microscopy system. (b) Raman spectra of Fe_3‐x_GeTe_2_/In_2_Se_3_ heterostructure as a function of gate voltage measured at 80 K. The four fitted Raman peak positions at zero bias are shown as black solid lines, while the peak positions with non‐zero gate bias are shown as gray dotted lines. Reproduced under terms of the CC‐BY license [92]. Copyright 2023, Eom et al., published by Springer Nature. (c) X‐ray photoemission electron microscopy (XPEEM) image of Fe_3_GeTe_2_/*α*‐In_2_Se_3_ heterostructure measured at 50 K with a field of view of 15 µm. The heterostructure region is shaded in pale blue. Reproduced under terms of the CC‐BY license [93]. Copyright 2024, Fujita et al., published by John Wiley and Sons. (d) Magnetic domain evolution of CuInP_2_S_6_/Fe_3_GaTe_2_ (CIPS/FGT) heterostructure under thermal cycling obtained by magnetic force microscopy. The black dashed line distinguishes the heterostructure region (left) and bare Fe_3_GaTe_2_ (right). The scale bar represents 1 µm. Reproduced with permission [94]. Copyright 2025, John Wiley and Sons.

Strain‐mediated mechanisms: As discussed in the 2D/3D multiferroic heterostructures, strain can serve as a powerful parameter for tuning magnetism also in 2D/2D multiferroic heterostructures. In‐plane strain generated by the ferroelectric layer enables the modulation of magnetic anisotropy and magnetic domain behavior. For instance, Eom et al. investigated the magnetic anisotropy of Fe_3‐x_GeTe_2_/In_2_Se_3_ vdW heterostructure [92]. The application of gate bias for both polarities significantly decreases the *H*_C_, as shown in Figure 3a, which can be ascribed to the decreased magnetocrystalline anisotropy induced by in‐plane tensile strain. The Raman spectra depending on gate voltage show decreased Raman peak positions (or redshift) of both In_2_Se_3_ and Fe_3‐x_GeTe_2_ regardless of the voltage polarity (Figure 3b). This indicates the voltage‐induced in‐plane tensile strain for both layers. In addition, hole doping caused by Fe deficiency is crucial for the magnetocrystalline anisotropy change by decreasing overall magnetocrystalline anisotropy energy and promoting the system to the tensile strain‐induced transition from out‐of‐plane to in‐plane magnetocrystalline anisotropy. Direct imaging of the magnetic domains provides complementary insight into the relationship between strain and magnetism. For instance, Fujita et al. reported a Fe_3_GeTe_2_/In_2_Se_3_ heterostructure, investigating the strain‐induced magnetic property change in terms of magnetic domains observed by X‐ray photoemission electron microscopy (XPEEM) [93]. The strain‐induced reduction of exchange stiffness lowers the domain wall formation energy, introducing more domain walls of the heterostructure, as shown in the XPEEM image (Figure 3c), which is supported by the reduced Curie temperature by ∼20 K. The reduction of the Curie temperature is attributed to the compressive lattice strain, corroborated by the density functional theory calculation. The motion of magnetic domain walls by ferroelectric polarization was systematically investigated by Zhao et al. using in situ magnetic force microscopy in a CuInP_2_S_6_/Fe_3_GaTe_2_ vdW heterostructure [94]. As depicted in Figure 3d, ferroelectric CuInP_2_S_6_ facilitates the formation of magnetic domain walls and hinders the wall's further motion. The ferroelectric polarization‐enhanced DMI is responsible for reducing the domain wall formation energy, driving the transition from coherent to incoherent magnetic reversal.

**FIGURE 4:**
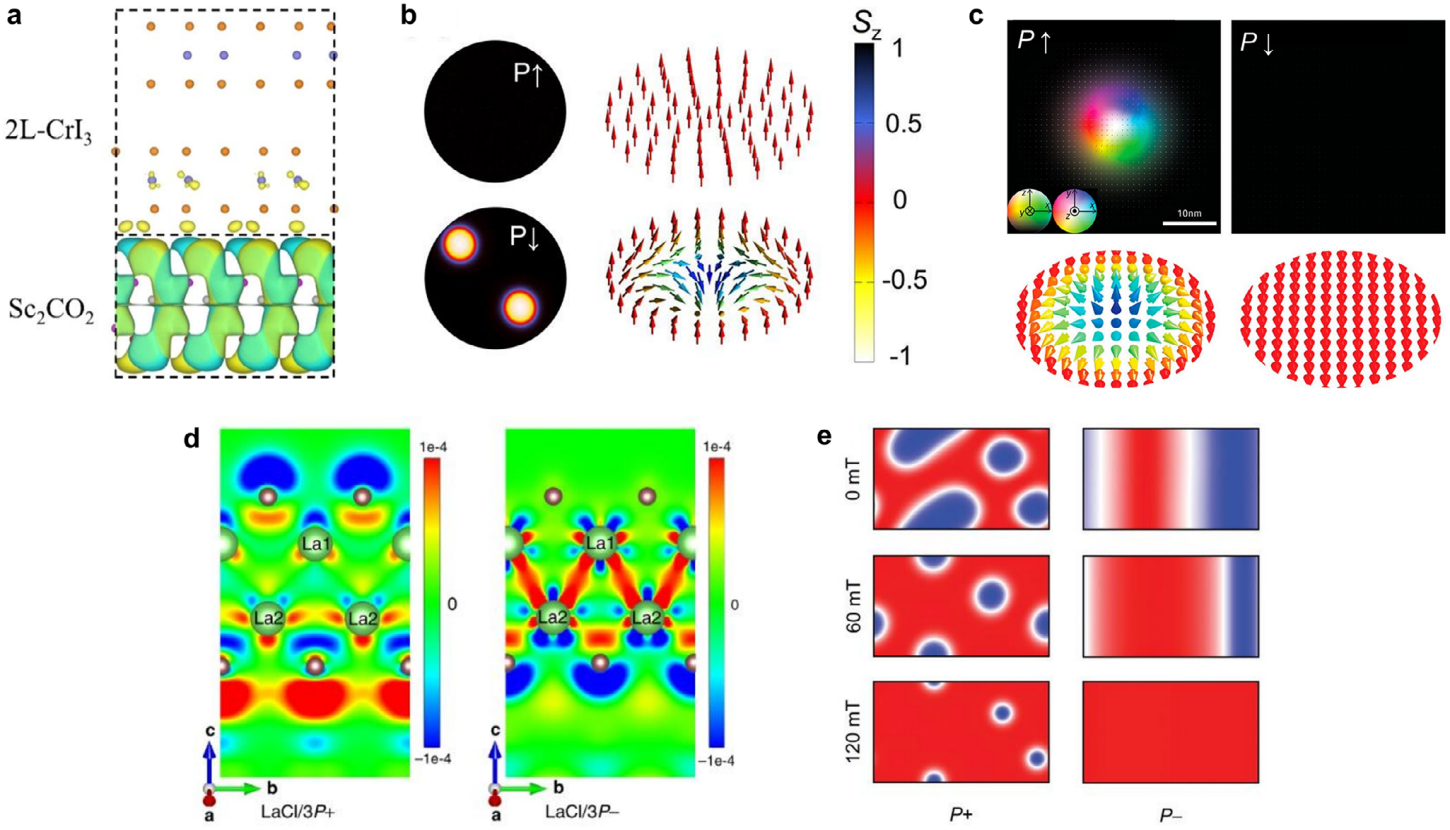
(a) Differential charge distribution of the bilayer CrI_3_/Sc_2_CO_2_ heterostructure calculated by the subtraction of the electron distribution of the down polarization from that of the up polarization of Sc_2_CO_2_. The isosurface value is 0.001 e/bohr^3^. The yellow color indicates the accumulation of electrons, whereas the cyan color indicates the loss of electron. Reproduced with permission [101]. Copyright 2020, American Chemical Society. (b) Real‐space spin textures of CrSeI/In_2_Te_3_ heterostructures with upward (P↑) and downward (P↓) polarizations. Reproduced with permission [112]. Copyright 2024, American Chemical Society. (c) Spin‐dynamics modeling of Fe_3_GeTe_2_/In_2_Se_3_ heterostructure for different ferroelectric polarizations. Reproduced with permission [113]. Copyright 2022, American Chemical Society. (d) Differential charge density on the LaCl side of the LaCl/In_2_Se_3_ heterostructure. Reproduced under terms of the CC‐BY license [114]. Copyright 2020, Sun et al., published by Springer Nature. (e) Spin textures with different polarizations of CuInP_2_S_6_ in WTe_2_/CrCl_3_/CuInP_2_S_6_ heterostructure by evolving the magnetic field. Reproduced with permission [105]. Copyright 2021, John Wiley and Sons.

Interfacial orbital hybridization: In addition to the strain, ferroelectric polarization can modify the orbital configuration of vdW ferromagnet across the interface, tuning the spin–orbit coupling and exchange interactions. In such multiferroic heterostructures, switching the ferroelectric polarization can reversibly change the magnetic anisotropy and the magnetic easy axis. For example, Li et al. investigated the electronic structure and magnetic properties of the FeBrI/In_2_S_3_ vdW heterostructure, and found that the MAE change is attributed to the orbital hybridization induced by the interfacial charge redistribution [95]. Wang et al. demonstrated the tuning of the magnetic easy axis by tunable orbital reconstruction via ferroelectric polarization in *α*‐RuCl_3_/CuInP_2_S_6_ vdW heterostructure [96]. Similarly, Jin et al. reported the ferroelectrically tunable orbital reconstruction and tunable easy axis in FeCl_3_/In_2_Se_3_ [97]. The reversible ferroelectric polarization switching in the heterostructure alters the MAE from 0.12 to −0.09 meV/Fe, indicating the change of magnetic easy axis from out‐of‐plane to in‐plane direction. By switching ferroelectric polarization, the MAE of the heterostructure can be enhanced by interfacial charge redistribution. For instance, Ji et al. reported the significant MAE enhancement in Cr_2_Si_2_Te_6_/In_2_Se_3_ vdW heterostructure by switching the ferroelectric polarization direction [98]. Strong ME coupling arises from the interfacial electron redistribution driven by the internal electric field associated with the broken spatial inversion symmetry of the ferroelectric layer. The interlayer distance between Cr_2_Si_2_Te_6_ and In_2_Se_3_ can also play a role in interfacial charge redistribution, where a shorter interlayer distance under the downward polarization allows more charges to transfer and makes MAE larger. Gong et al. studied the strong ME effect of Cr_2_Ge_2_Te_6_/In_2_Se_3_ heterostructure via first‐principles calculations, where the magnetic anisotropy of Cr_2_Ge_2_Te_6_ is flipped between in‐plane and out‐of‐plane orientations by reversal of ferroelectric polarizations of In_2_Se_3_ [32]. Detailed analysis revealed that the interfacial hybridization causes the interlayer ME coupling. Interestingly, In_2_Se_3_ becomes magnetized due to the proximity to Cr_2_Ge_2_Te_6_, demonstrating the unique system of dual interlayer and intralayer multiferroicity. Ferroelectric polarization can also toggle the sign of interlayer exchange in layered magnets. For example, Cheng et al. investigated the interlayer magnetic coupling of CrI_3_/In_2_Se_3_ heterostructure via first‐principles calculations [99]. Non‐volatile control of the ferroelectric polarization in In_2_Se_3_ enables the reversible switching of the interlayer magnetism in CrI_3_ between ferromagnetic and antiferromagnetic coupling. Wu et al. demonstrated the nonreciprocal and non‐volatile tuning of magnetic coercivity of CrI_3_/In_2_Se_3_ vdW heterostructure, where the amount of charge redistribution depends on the ferroelectric polarization direction [100]. The spin‐flip fields of antiferromagnetic CrI_3_ on ferroelectric In_2_Se_3_ are smaller than those on paraelectric hexagonal boron nitride (hBN), indicating enhanced ferromagnetic coupling at the presence of ferroelectric polarization. Lu et al. also observed the tunable ferromagnetic and antiferromagnetic states in bilayer CrI_3_/Sc_2_CO_2_ heterostructure [101]. Electron transfer from Sc_2_CO_2_ to CrI_3_ for the up polarization state, as shown in the differential charge distribution diagram in Figure 4a, switches the interlayer magnetic coupling from antiferromagnetic to ferromagnetic states. Given that the small energy difference between the interlayer ferromagnetic and antiferromagnetic orderings (∼0.37 meV), for a band gap reduction of 70 meV for ferromagnetic state compared to antiferromagnetic state, electron doping density of 0.01 e/unit cell (critical doping density to realize antiferromagnetic to ferromagnetic phase transition) can provide about 0.7 meV energy gain of the antiferromagnetic state than that of the ferromagnetic state, rendering the energetic preference of the ferromagnetic ground state. Beyond the conventional ferromagnets and antiferromagnets, polarization‐controlled interfacial band rearrangement can stabilize more exotic magnetic orders. Wu et al. introduced the interfacial ME coupling via interfacial band rearrangement of the MnPTe_3_/In_2_Se_3_ heterostructure [102]. Their first‐principles calculations reveal that the ferroelectric polarization of In_2_Se_3_ induces the transition of MnPTe_3_ from antiferromagnetism to altermagnetism, suggesting the possibility of reversible electrical control of spin‐splitting. The built‐in field from interfacial charge transfer alters the dominant valence band maximum contribution between the magnetic and ferroelectric layers, enabling control of spin‐splitting near the Fermi level.

#### 3.2.2 Emergent Spin Textures

Magnetic skyrmions are topologically protected spin textures exhibiting a whirling real‐space configuration [103], and they are regarded as promising information carriers for next‐generation memory and logic, such as racetrack devices [104, 105]. DMI is known to be central to the formation, stabilization, and manipulation of skyrmions, and emerges in 2D magnets with broken inversion symmetry [106, 107, 108]. Recently, skyrmion and its motion when applying electric current have been observed in 2D Fe_3_GeTe_2_ [109, 110], suggesting that 2D magnetic materials can serve as a skyrmion medium. For vdW heterostructures, the ease of interfacial engineering provides a flexible route to induce DMI via Rashba spin–orbit coupling at the interface [105]. Carrier reconstruction at the heterointerface also provides additional pathways to mediate the magnetic interactions, as discussed previously.

In vdW 2D multiferroic heterostructures, the influence of ferroelectric polarization on magnetism and related spin textures to control the skyrmion formation and annihilation has been a key question. The orbital hybridizations within the ferromagnetic/ferroelectric heterostructures can affect the skyrmion dynamics. For example, Han et al. presented a tunable total polarization of CrSeTe/In_2_Se_3_ heterostructure by switching the polarization direction of In_2_Se_3_ [111]. They observed significant magnetic anisotropy between Se or Te adjacent to In_2_Se_3_ (CTS/IS and CST/IS, respectively), stemming from the distinct *p_x_*–*p_y_* and *p_z_*–*p_y_* orbital hybridizations of the chalcogen atoms. By conducting spin dynamics simulations, the downward polarization in In_2_Se_3_ favored skyrmion formation, demonstrating skyrmion control by ferroelectric polarization. Wang et al. demonstrated the switching between magnetic skyrmion and high‐temperature ferromagnetic states of CrSeI/In_2_Te_3_ heterostructure by first‐principles studies [112]. Intrinsic magnetic skyrmion forms when the ferroelectric polarization of In_2_Te_3_ is downward, as shown in Figure 4b. The examination of orbital‐resolved MAE reveals that the positive MAEs are attributed to the orbital hybridization between Cr and I atoms, where the MAE difference depending on polarization direction is contributed by I atoms.

DMI can also play a vital role in polarization‐induced skyrmion control. Huang et al. investigated the creation and annihilation of skyrmions controlled by ferroelectric polarization in Fe_3_GeTe_2_/In_2_Se_3_ heterostructure [113]. The band structure of the heterostructure resembles that of freestanding Fe_3_GeTe_2_, suggesting negligible orbital hybridization across the interface due to the weak vdW interactions. Instead, based on first‐principles calculations of magnetic parameters and atomistic spin‐dynamics modeling, DMI is responsible for reversible skyrmion creation and annihilation, controlled by the ferroelectric polarization (Figure 4c). Sun et al. observed the magnetic skyrmions in the bimerons form, the usual form of skyrmions in an easy‐plane 2D magnet, generated by the broken inversion symmetry of ferromagnets by ferroelectric polarization [114]. In the LaCl/In_2_Se_3_ heterostructure, charge redistribution of LaCl occurs to screen the polarization field induced by the ferroelectric polarization of In_2_Se_3_. As the differential charge density distributions show, electronic reconfiguration occurs on the LaCl side and changes the exchange coupling constant, rendering a variation of DMI on the LaCl side (Figure 4d). They demonstrated the feasibility of non‐volatile control of magnetism and skyrmions in vdW 2D heterostructures. Liu et al. theoretically developed the LaX_2_ (X = Cl, Br, I)/In_2_Y_3_ (Y = S, Se, Te) system [115]. Their first‐principles calculations show that magnetic frustration in the ferromagnetic layers can be modulated by the direction of ferroelectric polarization, enabling the control of topological spin textures. The electronic properties associated with ferroelectric polarization are explained by band alignment and interfacial charge transfer. Finally, Sun et al. observed a skyrmion‐bimeron‐ferromagnetic phase transition cycle in a WTe_2_/CrCl_3_ heterostructure under a perpendicular magnetic field [105]. Placing them on a ferroelectric CuInP_2_S_6_ monolayer, skyrmionic bubbles emerge in the up polarization state, whereas the down polarization state is a striped state (Figure 4e). Under the magnetic field of 120 mT, the skyrmion morphology can be controlled by switching the ferroelectric polarization. This is attributed to the polarization‐induced electronic reconstruction at the interface of WTe_2_/CrCl_3_ and CuInP_2_S_6_, as shown in the differential charge density distribution and the integration of the densities in the *xy*‐plane. For the up polarization state, the Cl ions contacting the ferroelectric layer possess much higher electron density than the down polarization state, producing a higher inversion asymmetry and thus a higher DMI is induced. These results underscore the potential of vdW 2D multiferroic heterostructures for non‐volatile, electric field control of skyrmion‐based spin textures.

### 3.3 Emergent vdW Multiferroic Devices

While ferromagnetic/ferroelectric vdW heterostructures appear to be a compelling route toward vdW multiferroics, several alternative strategies have recently emerged for realizing multiferroic properties. In this section, we discuss vdW multiferroic tunnel junctions (MFTJs), which exploit the high tunneling efficiency enabled by atomically clean and dangling bond‐free interfaces of vdW materials, as well as antiferromagnetic and altermagnetic‐based multiferroics. We further discuss twist‐controlled multiferroic heterostructures, in which periodic moiré potentials render emergent multiferroic phenomena and ME functionalities.

#### 3.3.1 VdW Multiferroic Tunnel Junctions

The emergence of MFTJs, by combining two different magnetization alignments of the ferromagnetic electrodes with a ferroelectric ultrathin film as the tunnel barrier, has shown promise in addressing the challenges faced by conventional silicon‐based semiconductor devices. MFTJs exhibit multiple nonvolatile resistance states by combining the tunnel magnetoresistance (TMR) effect from magnetic tunnel junctions and the tunnel electroresistance (TER) effect from ferroelectric tunnel junctions. Multi‐resistance MFTJs represent a unique condensed matter system enriched by the complex interplay between ferroic polarizations and electron tunneling, governed by diverse microscopic mechanisms, including polarization‐dependent band alignment [116], spin‐dependent transmission [117, 118] and ME coupling between different ferroic orders [119]. Traditional MFTJs typically have relied on epitaxial oxide multilayers [118, 120]. Since the tunneling efficiency is extremely susceptible to lattice and interface imperfections, oxide multilayers with unavoidable structural complexities such as oxygen vacancies, interfacial defects, and dangling bonds hampered the exploration of high‐performance MFTJs [121]. In this viewpoint, 2D vdW materials with uniform surfaces and free of dangling bonds are attractive candidates to build vdW MFTJs with a sandwiched structure. For example, Xie et al. demonstrated all‐vdW MFTJs using asymmetric electrodes with TER of 10^6^% and an ON‐state current density of 10^4^ A cm^−2^ in Fe_3_GeTe_2_/In_2_Se_3_/Fe_5_GeTe_2_ structure, both of which are two orders of magnitude higher than the highest values ever reported in MFTJs [121]. They further demonstrated room‐temperature operation using Fe_3_GaTe_2_ electrode, which is crucial for practical applications of vdW MFTJs for multistate memory. Also, high resistance‐area (RA) product of the conventional oxide‐based systems (kΩ·µm^2^ [122] to MΩ·µm^2^ [119, 120, 123, 124]) due to the critical thickness (few nm) of ferroelectric materials and relatively large bandgap can be effectively overcome by using vdW 2D materials. Owing to the robust ferroelectric polarization down to the monolayer limit (e.g., In_2_Se_3_), vdW 2D materials can efficiently work as an ultrathin tunnel barrier in MFTJs [125]. Thus, the RA product of vdW MFTJs should be much lower than the conventional oxide‐based MFTJs due to 2D ferroelectricity and its narrow bandgap. For example, Su et al. demonstrated the distinguished four resistance states of PtTe_2_/Fe_4_GeTe_2_/*α*‐In_2_Se_3_/Fe_3_GeTe_2_/PtTe_2_ MFTJs, with all states having RA product lower than 1 Ω·µm^2^ [125]. A small bandgap of monolayer *α*‐In_2_Se_3_ (∼0.8 eV) renders the Fermi energy crossing the conduction bands of *α*‐In_2_Se_3_, leading to the small RA product. Overall, vdW MFTJs with high interfacial quality, multiple resistance states, and ultralow RA products, making them powerful candidates for future spintronics applications.

#### 3.3.2 Antiferromagnetic and Altermagnetic vdW Multiferroics

As a promising alternative to ferromagnets, antiferromagnetic materials have drawn increasing attention owing to their faster spin dynamics and low sensitivity to stray magnetic fields, advantageous for spintronics applications [126]. However, effective electrical control remains a critical challenge in the field of antiferromagnetic spintronics [127]. Realization of vdW multiferroics via ferroelectric control of antiferromagnetic order is particularly interesting for energy‐efficient and ultrafast spintronics.

As briefly discussed in the introduction of this section, sliding ferroelectricity found in vdW 2D bilayers is an intriguing avenue to realize 2D ferroelectricity. Strikingly, the vertical ferroelectric switching is induced by in‐plane interlayer sliding without any requisite vertical ion displacements, distinct from the conventional ferroelectrics. The theoretical model and experimental investigations of sliding ferroelectricity are comprehensively discussed in reference [128]. This unique mechanism gives rise to ultralow switching barriers (approximately millielectron volts per formula unit), enabling high‐speed data writing with low energy. Therefore, searching for 2D antiferromagnetic materials with remarkable sliding ferroelectricity and ME coupling is of great significance to realize high‐speed, low‐power spintronic devices [128]. Indeed, unique ME coupling can arise from the sliding ferroelectricity. For two ferromagnetic monolayers that are antiferromagnetically coupled, inequivalent stacking may exhibit both sliding ferroelectricity and uncompensated magnetic moments. For example, multiferroic bilayer VS_2_ composed of two ferromagnetic monolayers is possible for VCM via interlayer sliding based on first‐principles calculations [129]. The interaction between interlayer ferrovalleys results in asymmetric energy splitting at the conduction band minimum and valence band maximum for the *K*_+_ and *K*_−_ valleys, which can be exchanged via ferroelectric switching or magnetic reversal for both layers. Similar sliding‐induced ME coupling is predicted for polar‐stacked MnSe [130], MnBi_2_Te_4_ [131], CrTe_2_ [132], and Fe_5_GeTe_2_ [133].

The potential of antiferromagnets can be further strengthened when the parity‐time‐reversal symmetry is broken, a regime that is found in a newly identified subclass of collinear antiferromagnets termed altermagnets. In contrast to conventional antiferromagnets, altermagnets exhibit antiferromagnetically coupled spin configuration with alternating sublattice symmetries, disrupting parity‐time‐reversal symmetry and generating a momentum‐dependent spin‐splitting of the electronic band structure [134]. Recent work shows the generation of electric polarization by altermagnetic Néel order, demonstrating the possibility of type‐II multiferroicity [135]. However, switching the Néel order is considered energy inefficient due to a large current density needed to produce spin‐transfer or spin–orbit torques [136, 137]. Ferroelectric control of altermagnetic splitting without altering the Néel vector, as proposed in materials such as MnP(S, Se)_3_ [132, 138], VP(S, Se)_3_ [132], and AgF_2_ [139], is considered a promising platform for energy‐efficient spintronics.

#### 3.3.3 Twist‐Controlled vdW Multiferroic Heterostructures

**FIGURE 5:**
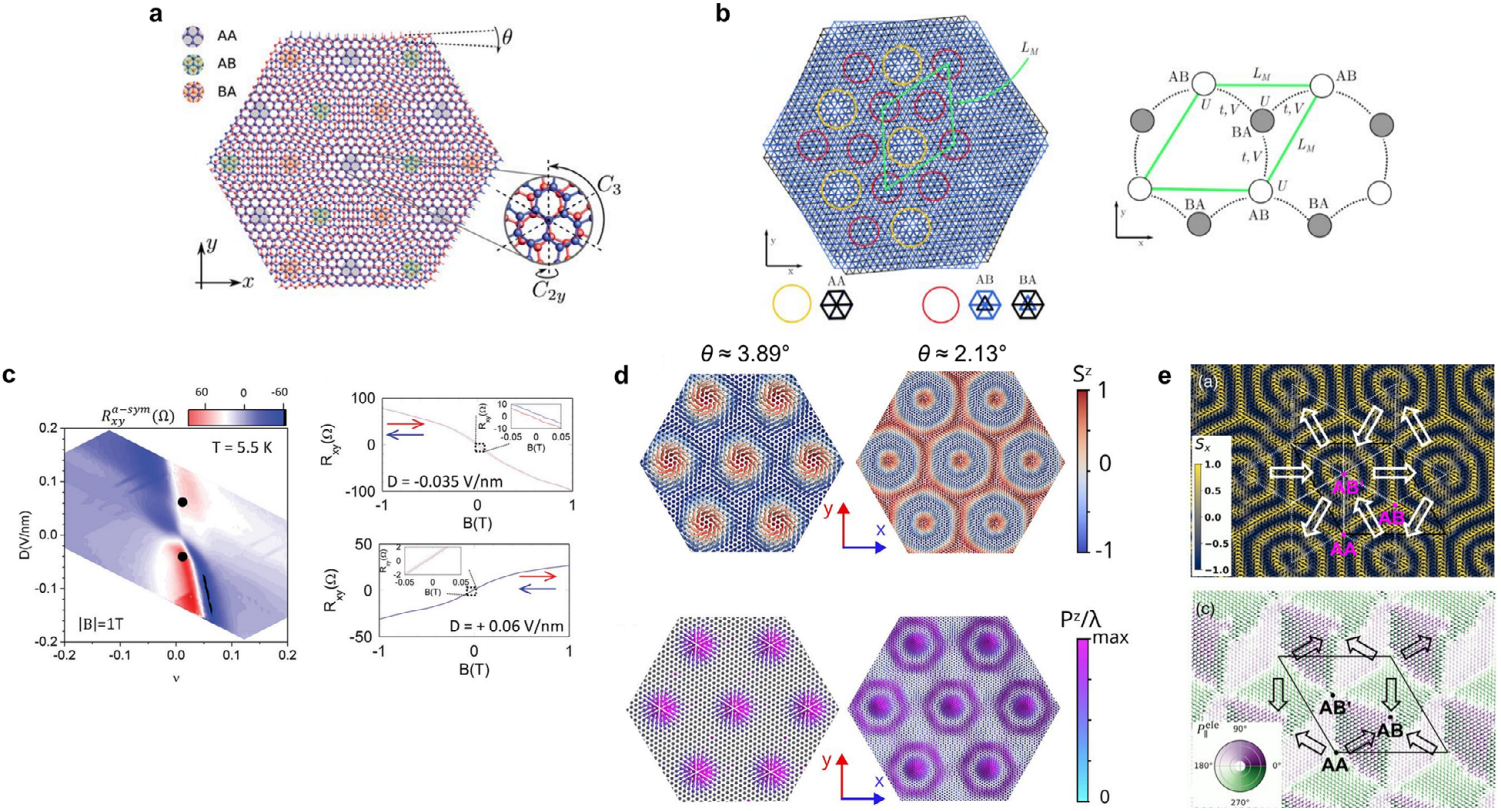
(a) Moiré superlattice of bilayer graphene formed with an interlayer twist angle *θ*. The local AA, AB, and BA stacking are indicated as gray, green, and orange regions. Reproduced with permission [146]. Copyright 2018, American Physical Society. (b) Twisted bilayer triangular lattice. Sites AB and BA are structurally equivalent and display a staggered honeycomb lattice with a moiré length *L*_M_. Moiré unit cell in green and AB (BA) sites are depicted as white (gray) circles. First‐neighbor hopping *t*, on‐site *U*, and first‐neighbor *V* Coulomb interactions are schematically shown. Reproduced under the terms of the CC‐BY license [151]. Copyright 2022, Haavisto et al., published by SciPost. (c) Left: 2D map of anti‐symmetrized Hall resistance as a function of moiré filling factor and displacement field. Right: Anomalous Hall resistance as a function of perpendicular magnetic fields. Insets show a magnified view of the encircled region to reveal the hysteresis. Reproduced with permission [153]. Copyright 2024, John Wiley and Sons. (d) Spin (top) and polarization (bottom) of a skyrmion and skyrmionium lattice in twisted bilayer NiI_2_. (twist angles *θ* ≈ 3.89° and *θ* ≈ 2.13° for left and right panels, respectively) The panels show the top layer of a twisted bilayer, with the bottom one featuring a magnetic configuration that is antialigned with the top, and an equivalent polarization profile. Reproduced under terms of the CC‐BY license [157]. Copyright 2024, Antão et al., published by American Chemical Society. (e) Top: Spin mapping in twisted bilayer NiI_2_ at *θ* = 2.13°. Black arrows indicate the direction and magnitude of in‐plane magnetization, while the background color is mapping the x‐direction spin (*S*_x_) component. Large white arrows indicate the local spiral wave vectors. Bottom: Electric polarization pattern generated by the spin texture. Small arrows indicate local polarizations between nearest Ni–Ni pairs, while color indicates their in‐plane orientation. Large black arrows denote net polarization directions in specific domains. Reproduced with permission [158]. Copyright 2025, American Physical Society.

Twist engineering of vdW 2D materials has recently emerged as a powerful approach for creating artificial electronic and structural phases [140, 141, 142, 143, 144, 145]. When two atomically thin layers are stacked together with a small relative twist, a moiré superlattice forms and imposes a long‐wavelength periodic potential on the electronic states. A representative example is twisted bilayer graphene, where a small twist angle produces a moiré pattern with an enlarged real‐space unit cell (Figure 5a) [146]. By controlling the twist angle between two identical or dissimilar vdW layers, the manipulation of symmetry and emergent behaviors such as artificial ferroelectricity and magnetism can be realized. For example, twisted bilayer hBN [81] or twisted transition metal dichalcogenides [147, 148] exhibit artificial ferroelectricity due to the broken inversion symmetry through the specific rotations between vdW bilayers. Furthermore, moiré superlattices with broken symmetries generated by specific twist angles exhibit correlation‐driven magnetic ordering, realizing the coexisting ferroelectric and magnetic order parameters [143, 149, 150]. A representative microscopic picture is provided by twisted TMD homobilayers, where the moiré potential flattens the low‐energy bands and localizes their Wannier states on a staggered honeycomb lattice described by an interacting Hamiltonian with hopping *t*, on‐site Coulomb interaction *U*, and nearest‐neighbor Coulomb interaction *V* (Figure 5b) [151]. At quarter‐filling, correlation‐driven spin polarization produces magnetic order, while nearest‐neighbor repulsion favors an imbalanced charge occupation between the two sublattices. Because the two sublattices are localized in opposite layers, this charge imbalance breaks inversion symmetry and generates a spontaneous out‐of‐plane electric polarization. The accompanying sublattice charge imbalance also renders the two sublattice magnetizations inequivalent, giving rise to coexisting ferrimagnetic and ferroelectric orders. In this intrinsic moiré multiferroic state, the magnetic and electric orders share the same charge distribution and are therefore intrinsically locked by strong ME coupling, allowing an applied electric field to tune the magnetization. The moiré length scale is essential to this mechanism, as it enlarges the effective lattice constant so that nearest‐neighbor repulsion between sublattices, or equivalently between layers, becomes sufficiently important to stabilize layer polarization together with the on‐site interaction. Complementary momentum‐space exact‐diagonalization studies of topological flat bands reach the same conclusion from a different starting point: long‐range Coulomb repulsion drives coexisting spin‐valley polarization, corresponding to ferromagnetism through broken time‐reversal symmetry, and layer polarization, corresponding to ferroelectricity through broken *C*_2y_ symmetry. This interaction‐driven multiferroic phase competes with a ferromagnetic Chern insulator, and the screening length set by the gate distance can tune the system between the two phases [152].

These intriguing phenomena under the interplays of lattice symmetries depending on twist angle would possibly extend to twist‐controlled multiferroic behavior. For example, Hassan et al. reported the multiferroicity of 3° twisted WSe_2_ at 5.5 K, exhibiting coexistence of ferroelectric and correlation‐driven ferromagnetic ordering [153]. The dual gate measurements of Hall resistance (*R_xy_*) reveal the presence of magnetic domains by the appearance of non‐zero anti‐symmetrized Hall resistance (*R_xy_* ^a‐sym^) near moiré filling fraction *ν* = 0 (Figure 5c, left). Also, the anomalous Hall effect with finite hysteresis at positive (0.06 V nm^−1^) and negative (−0.035 V nm^−1^) displacement fields (Figure 5c, right) indicates ferromagnetic characteristics, which is induced by the strong electronic correlation providing a localization effect and topological bands due to non‐zero Berry curvature. They further showed that the moiré superlattices undergo commensurate‐incommensurate transitions for the twist angle of *θ* ≥ 4°, accompanied by vanished ferroelectricity due to the rigid domain walls, underscoring the critical role of lattice relaxation and commensurability. Multiferroicity can be achieved by twisting magnetic monolayers, while sliding allows polarization direction and magnetic states to be switched via small interlayer displacements. For example, twisted NiI_2_ and CrX_3_ (X = Cl, Br, I) can exhibit multiferroicity with emergent spin textures. Recently, type‐II multiferroic NiI_2_ with spin‐spiral order has been identified as odd‐parity (*p*‐wave) magnetism [154] and strain‐mediated control of spin‐spiral multiferroic phase [155]. This highlights the interplay between multiferroicity, noncollinear magnetism, and unconventional spin‐split electronic states in spin‐spiral vdW materials [156], providing a unique platform for developing ultrafast, energy‐efficient spintronic devices. Recent work of the synthesis methods of NiI_2_ will be briefly discussed at Section 4.1. Antão et al. demonstrated the electrically tunable moiré magnetisms in twisted bilayers of the multiferroic NiI_2_ [157]. Two distinct topological magnetic phases of commensurate cases, i.e., twist angle *θ* ≈ 3.89° and *θ* ≈ 2.13°, show the emergent polarization associated with skyrmion and skyrmionium lattices in the top layer of the twisted NiI_2_ system (Figure 5d). The strong ME coupling in the twisted multiferroic enables the manipulation of skyrmion lattice phases using an external electric field. Zhu et al. observed that lattice relaxation is crucial for polar‐magnetic topologies for twisted bilayer NiI_2_ [158]. At the twist angle *θ* = 2.13°, the spin pattern achieves its highest order, forming a *C*_3_‐symmetric domain around AB′ stacking (Figure 5e, top). Further calculation at the twist angle *θ* = 2.13° with rigid layers reveals that the distinctive spin spiral pattern vanishes without atomic relaxation. Electric polarization in the *xy* plane is predicted (Figure 5e, bottom) with oriented perpendicular to the in‐plane spiral wave vector. Fumega and Lado investigated the twisted chromium halide CrX_3_ (X = Cl, Br, I) bilayers using first‐principles and effective spin Hamiltonians [159]. In combination with spin–orbit coupling, this local non‐collinearity produces an emergent electrical polarization in the moiré domains. Taken together, these studies indicate that twisted vdW heterostructures provide a versatile route to realize the concurrent ferroic ordering, where ferroelectricity and magnetism arise from the same twist‐engineered electronic and magnetic landscape, offering a promising pathway toward multiferroics. Overall, the direct comparison of device parameters for practical applications, including operating temperature, ME coupling coefficient, switching energy, and ME coupling mechanisms of oxide‐based systems (Section 2) and vdW systems (Section 3) is summarized in Table 1.

**TABLE 1:** Benchmarking key performances of oxide and vdW multiferroic heterostructures for the magnetoelectric coupling effect. The magnetic fields are in mT (1 mT ≈ 10 Oe).

Device structure	Operating temperature	Magnetoelectric coupling coefficient	Switching energy	Coercive magnetic field (electric field)	Magnetoelectric coupling mechanisms	Reference
3D/3D						
PMN‐PT/Ni‐Mn‐In	R.T.	∼4.1 V cm^−1^ Oe^−1‡^	—	—	Strain‐mediated interfacial coupling	[63]
FeGa/MgO/PMN‐PT	R.T.	∼1.5 × 10^−5^ S m^−1^	—	∼4 mT (3 kV cm^−1^) ∼9 mT (−3 kV cm^−1^)	Magnetoelastic strain	[160]
Co_2_FeSi/PMN‐PT	R.T.	∼1.0 × 10^−5^ S m^−1^	—	—	Piezoelectric strain	[161]
Ta/Co/BiFeO_3_/SrRuO_3_	5–300 K	—	—	1–6 mT (10 V)	Magneto‐ionic effect	[62]
BiFeO_3_/La_0.7_Sr_0.3_MnO_3_	10–350 K	—	—	—	Electronic correlation	[64]
AlScN/Ta_2_O_5_/Co_0.2_Fe_0.6_B_0.2_/MgO	—	—	—	5.7–14.1 mT (20 V)	Minority‐spin band structure	[66]
2D/3D						
Fe_3_GaTe_2_/PMN‐PT	297 K	—	—	∼90 mT (0 kV/cm^−1^) ∼30 mT (12 kV cm^−1^)	Piezoelectric strain	[89]
P(VDF‐TrFE)/Cr_2_Ge_2_Te_6_	4 K	—	—	5 mT (5 V)	Interfacial orbital hybridization	[44]
P(VDF‐TrFE)/Fe_3_GaTe_2_	330 K	(negative) 8.7 × 10^−6 ^S m^−1^ (positive) 4.2 × 10^−6 ^S m^−1^	10.125 aJ	55 mT (−90 V)	Interfacial orbital hybridization	[91]
2D/2D						
Fe_3‐x_GeTe_2_/In_2_Se_3_	67 K	—	—	30 mT (0 V) 5 mT (5 V) 18 mT (−5 V)	Piezoelectric strain	[92]
Fe_3_GeTe_2_/In_2_Se_3_	50–200 K	—	—	—	Piezoelectric strain	[93]
CuInP_2_S_6_/Fe_3_GaTe_2_	293 K	—	—	6.5 mT (0 V) 2.0 mT (±5 V)	Dzyaloshinskii–Moriya interaction	[94]
CuCrP_2_S_6_/Fe_3_GeTe_2_	153 K	—	—	∼22 mT (5 V) ∼7 mT (−5 V)	Interfacial orbital hybridization	[162]
CrI_3_/In_2_Se_3_	10 K	—	—	5.6 mT (0 V) 10.0 mT (10 V)	Interlayer exchange coupling	[100]
Twisted bilayer WSe_2_	5.5 K	—	—	—	Electronic correlation	[153]

‡ Direct magnetoelectric voltage coefficient.

## 4 Fabrication Strategies for High‐Performance 2D Multiferroic Heterostructures

The diverse material platforms and architectures discussed in the previous section, ranging from 2D ferromagnetic/ferroelectric heterostructures to twisted moiré superlattices, offer promising practical routes toward realizing beyond‐classical multiferroics. However, bridging the gap between these theoretical predictions and experimental realization depends critically on fabrication fidelity. Whereas conventional oxide heterostructures are often constrained by lattice mismatch and strain clamping, the performance of vdW multiferroic devices is predominantly governed by interfacial quality and cleanliness [43, 163, 164]. Since ME coupling in these systems is mediated by short‐range interactions such as charge transfer, exchange proximity, or electrostatic modulation, even atomically thin contaminants or subtle structural disorder can critically suppress the desired multiferroic response [165, 166, 167].

In this section, we review state‐of‐the‐art fabrication methodologies required to construct high‐quality 2D multiferroic heterostructures. We focus on technical challenges and solutions in three key aspects: (1) the synthesis of pristine 2D building blocks, highlighting phase engineering via epitaxial growth; (2) advanced assembly techniques designed to minimize interfacial residues and achieve precise crystallographic alignment; and (3) interface engineering strategies to ensure environmental stability and enhance coupling efficiency. Understanding these processing–structure–property relationships is essential for transitioning 2D multiferroics from laboratory curiosities to practical low‐power logic and memory devices [33].

### 4.1 Synthesis Paradigms for Phase Engineering in 2D Ferroics

Constructing high‐performance 2D multiferroic heterostructures fundamentally begins with the controlled synthesis of their constituent layers. To achieve robust ME coupling at the interface, constituent layers should exhibit intended ferromagnetic or ferroelectric order with high phase purity and minimal extrinsic disorder. Currently, two distinct approaches are employed depending on the target application: mechanical exfoliation and bottom‐up synthesis. Mechanical exfoliation from bulk crystals remains the gold standard for fundamental studies, offering pristine crystallinity essential for verifying ME coupling mechanisms. However, small µm‐scale size of the flake, low throughput, and low yield of the exfoliation hamper the practical applications of low‐power devices. Consequently, recent efforts of growth methods have shifted toward vapor‐phase growth, such as molecular beam epitaxy (MBE) [168] and chemical vapor deposition (CVD) [169]. Including scalability, these growth techniques offer decisive advantages: the ability to stabilize thermodynamically metastable phases and to selectively introduce defects inducing ferroic orders [170].

Beyond the synthesis of individual ferroic layers, recent efforts have begun to address the direct preparation of 2D multiferroic vdW systems. The controlled growth of NiI_2_ down to the monolayer limit, noted above for its helimagnetic order [35], demonstrated that an intrinsic type‐II multiferroic state—in which a spin spiral generates electric polarization—can survive in an as‐grown single 2D material without requiring heterostructure assembly. Related experimental efforts have established vapor‐phase deposition as a scalable route to NiI_2_ crystals [171] and have extended intrinsic 2D multiferroicity beyond nickel halides, with few‐layer CuCrP_2_S_6_ exhibiting coexisting room‐temperature ferroelectricity and low‐temperature ferromagnetic order [172]. In parallel, bottom‐up growth of complementary ferroic building blocks, such as wafer‐scale MBE‐grown Fe_3_GeTe_2_ [173] and large‐area monolayer *α*‐In_2_Se_3_ epitaxially grown on graphene at low temperature [174], provides a materials basis for grown ferromagnet/ferroelectric vdW stacks. The feasibility of transfer‐free all‐vdW growth has also been demonstrated in Fe_3_GeTe_2_‐based heterostructures [175], suggesting a practical route toward cleaner multiferroic interfaces, although systematic growth of fully ferromagnetic/ferroelectric vdW stacks remains an important future direction. Such transfer‐free approaches are directly relevant to the ME device functionality observed in Fe_3‐x_GeTe_2_/In_2_Se_3_ heterostructures discussed in Section 3 [92], because polymer residues and interfacial disorder can screen polarization fields and suppress short‐range ME coupling. A comprehensive account of synthesis progress and remaining challenges for 2D vdW ferroic materials and their heterostructures can be found in reference [176].

**FIGURE 6:**
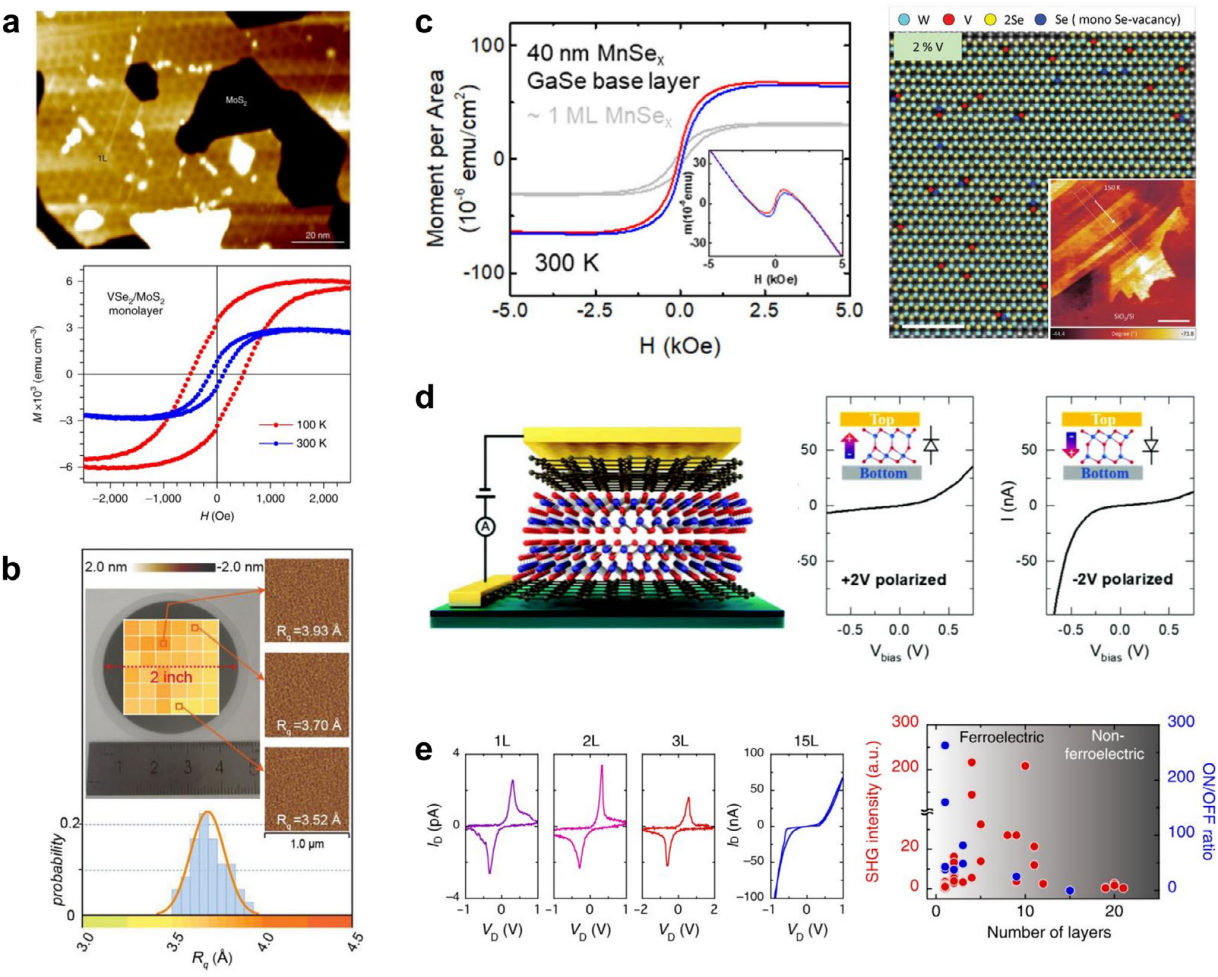
Synthesis paradigms for tailoring 2D ferroic orders and constructing pristine heterostructures. (a) Stabilization of metastable and wafer‐scale ferromagnets. STM image of monolayer 1T‐VSe_2_ grown via MBE, demonstrating the stabilization of a metastable ferromagnetic phase. Reproduced with permission [177]. Copyright 2018, Springer Nature. (b) Wafer‐scale CVD‐grown CrTe_2_ films on SiO_2_/Si, showing thickness‐dependent magnetic behavior. Reproduced with permission [178]. Copyright 2023, Springer Nature. (c) Defect‐mediated magnetism. Left: Magnetic hysteresis loop of epitaxial manganese selenide (MnSe_x_) thin films, demonstrating that precise stoichiometry control (Se‐deficiency) is critical for inducing room‐temperature ferromagnetism. Reproduced with permission [179]. Copyright 2018, American Chemical Society. Right: STEM image of V‐doped WSe_2_, with the inset displaying magnetic force microscopy (MFM) results that verify room‐temperature ferromagnetism induced by substitutional doping. Reproduced under terms of the CC‐BY license [180]. Copyright 2020, Wiley‐VCH GmbH. (d) Realizing functional 2D ferroelectrics. Demonstration of room‐temperature ferroelectricity and a switchable diode effect in exfoliated *α*‐In_2_Se_3_ layers. Reproduced with permission [181]. Copyright 2018, Royal Society of Chemistry. (e) Second‐harmonic generation (SHG) measurements of monolayer SnS, demonstrating robust in‐plane ferroelectricity at room temperature. Reproduced under terms of the CC‐BY license [182]. Copyright 2020, Springer Nature.

In the realm of 2D ferromagnetism, controlled epitaxial growth is particularly powerful for stabilizing metastable phases. A representative example is the monolayer 1T‐VSe_2_ reported by Bonilla et al. [177] They grew 1T‐VSe_2_ by MBE on vdW substrates such as highly oriented pyrolytic graphite (HOPG) and MoS_2_, with the observation of robust room‐temperature ferromagnetism in the monolayer limit, in clear contrast to the non‐magnetic bulk phase (Figure 6a). Building on this concept of phase‐stabilizing epitaxy, recent efforts have extended to the controlled synthesis toward materials with coupled ferroic functionalities. For instance, Song et al. achieved the controlled synthesis of monolayer NiI_2_ demonstrating that carefully optimized growth conditions can stabilize helimagnetic orders even in the 2D limit [35]. Furthermore, Liu et al. reported the wafer‐scale CVD growth of CrTe_2_, showing that phase‐controlled synthesis of 2D ferromagnets is now compatible with industrial scales (Figure 6b) [178]. Scalable thin film fabrication and defect control are key themes for practical applications. Liu et al. demonstrated wafer‐scale Fe_3_GeTe_2_ synthesis [173], and O'Hara et al. revealed that controlling the stoichiometry (such as Se‐deficiency) is critical for inducing room‐temperature ferromagnetism in epitaxial MnSe_x_ thin films (Figure 6c, left) [179]. This concept was further extended by Yun et al., who utilized substitutional doping (e.g., V‐doped WSe_2_) to realize room‐temperature ferromagnetic semiconductors (Figure 6c, right) [180].

Besides, the realization of functional 2D ferroelectrics is also important. Wan et al. utilized mechanically exfoliated ferroelectric *α*‐In_2_Se_3_ layers to demonstrate a switchable diode effect governed by polarization flipping (Figure 6d) [181]. Regarding ionic ferroelectrics, Liu et al. realized room‐temperature ferroelectricity in CuInP_2_S_6_ ultrathin flakes, confirming the potential of vdW ferroelectrics [39]. For group‐IV monochalcogenides, Chang et al. utilized MBE to increase the Curie temperature of SnTe via strain [74]. More recently, Higashitarumizu et al. achieved robust room‐temperature in‐plane ferroelectricity in monolayer SnS, experimentally validating the theoretical prediction of ferroelectricity in the 2D limit (Figure 6e) [182].

### 4.2 Assembly Techniques for Constructing Clean Interfaces

Once the constituent layers are prepared, the assembly process plays a pivotal role in determining the final device performance. Unlike covalently bonded 3D heterostructures, vdW 2D stacks are held together by relatively weak vdW forces, making the interface highly susceptible to trapped adsorbates and residues. Conventional wet transfer methods often leave polymer residues or solvent molecules at the interface, which can function as dielectric dead layers and scattering centers.

**FIGURE 7:**
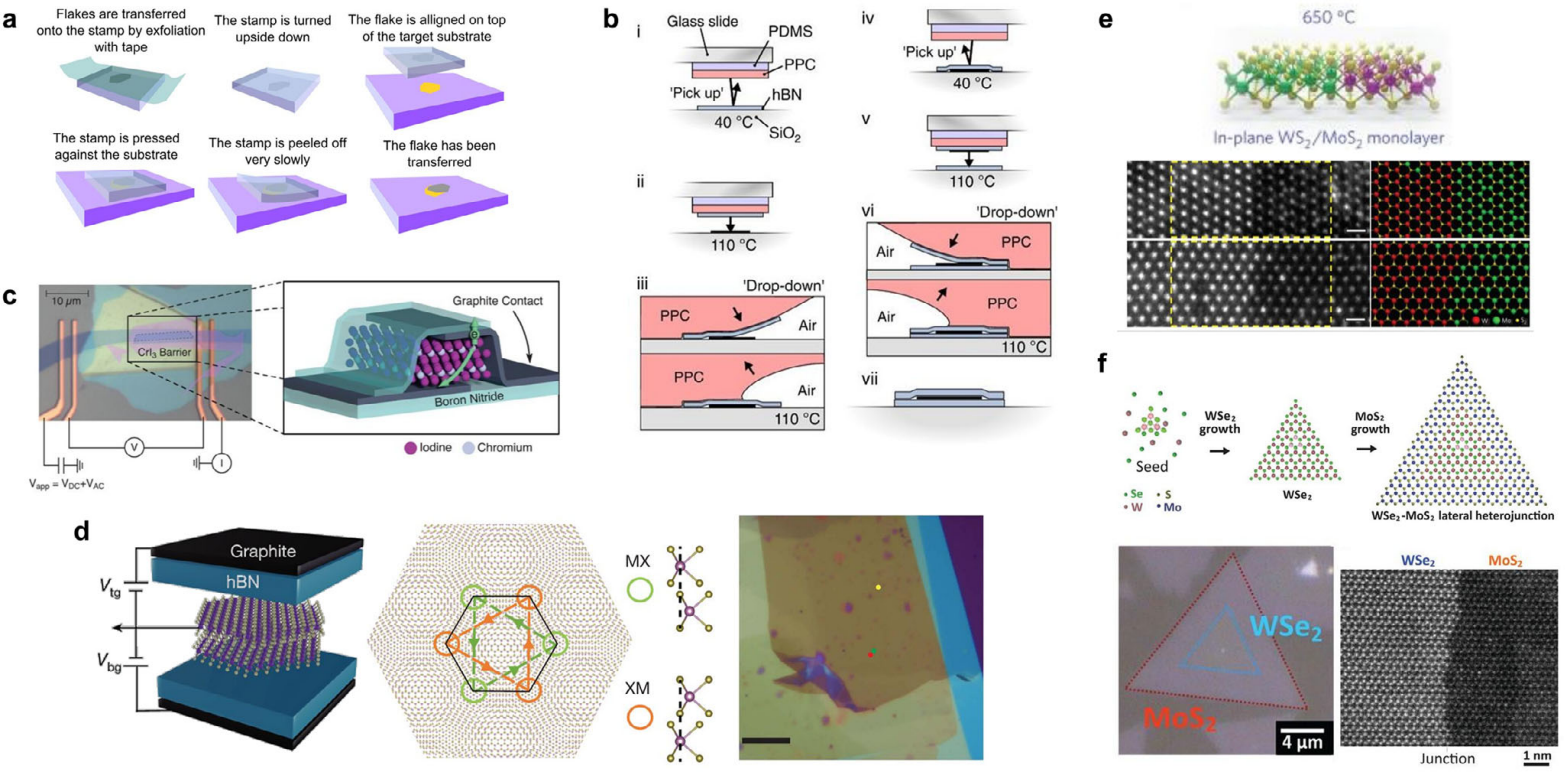
Advanced assembly techniques for constructing high‐quality vdW interfaces. (a) Deterministic dry transfer. Schematic of the all‐dry viscoelastic stamping method using PDMS to transfer 2D flakes, ensuring precise alignment without solvent contamination. Reproduced with permission [183]. Copyright 2014, IOP Publishing. (b) Interface cleaning via thermal management. The “hot pick‐up” technique, where the thermal expansion of the polymer stamp during lamination squeezes out interfacial bubbles and residues, resulting in atomically clean areas. Reproduced under terms of the CC‐BY license [184]. Copyright 2016, Springer Nature. (c) Assembly of air‐sensitive heterostructures. Schematic of the fabrication process for CrI_3_ tunnel junctions performed inside a glovebox to prevent degradation, enabling the observation of intrinsic tunneling magnetoresistance. Reproduced with permission [185]. Copyright 2018, American Association for the Advancement of Science. (d) Precision alignment for twistronics. Optical images illustrating the “tear‐and‐stack” technique applied to MoTe_2_, creating high‐quality twisted homobilayers that host fractional quantum anomalous Hall states. Reproduced with permission [186]. Copyright 2023, Springer Nature. (e) Direct growth of vdW heterostructures. Plan‐view STEM image of vertically stacked WS_2_ on MoS_2_, displaying moiré fringes that indicate a pristine interface. Reproduced with permission [187]. Copyright 2014, Springer Nature. (f) Image and schematic of a lateral WSe_2_–MoS_2_ heterojunction synthesized via one‐step epitaxy. Reproduced with permission [188]. Copyright 2015, American Association for the Advancement of Science.

To mitigate these issues, deterministic dry viscoelastic stamping and vacuum stacking techniques have been developed. The “pick‐up” technique using polydimethylsiloxane (PDMS), established by Castellanos‐Gomez et al. [183], remains the de facto standard for prototyping novel vdW heterostructures (Figure 7a). To further enhance cleanliness, Pizzocchero et al. introduced the “hot pick‐up” technique, utilizing thermal expansion to squeeze out contaminants (Figure 7b) [184]. For specific needs, Zomer et al. optimized a fast pick‐up technique using polypropylene carbonate (PPC) to ensure high‐quality heterostructures [189], and Frisenda et al. provided a comprehensive overview of these deterministic assembly methods [190].

Due to the generally air‐sensitive characteristics of vdW 2D materials, vacuum or inert‐gas assembly is mandatory. Klein et al. demonstrated the fabrication of CrI_3_ tunnel junctions in a glovebox, showing that vacuum stacking is a prerequisite for observing tunneling magnetoresistance (Figure 7c) [185]. In the context of “twistronics,” the utilization of the “tear‐and‐stack” method has extended beyond graphene; Cai et al. recently employed this technique to fabricate pristine twisted MoTe_2_ homobilayers, leading to the groundbreaking observation of fractional quantum anomalous Hall states (Figure 7d) [186]. Finally, to approach intrinsic coupling limits, the automated cleaning protocols by Purdie et al. [191] and hBN encapsulation benchmarks by Wang et al. [192] have been the essential references for minimizing interfacial disorder.

Ultimately, beyond mechanical stacking, direct growth strategies have emerged to form pristine interfaces intrinsically free from transfer‐related contaminants. Gong et al. pioneered the vertical growth of WS_2_ on MoS_2_, creating atomically sharp interfaces without polymer residues (Figure 7e) [187]. Recent progress includes the work of Li et al., who reported the direct synthesis of lateral WSe_2_‐MoS_2_ heterostructures, providing a platform for in‐plane coupling (Figure 7f) [188]. Thus, transitioning from exfoliation and transfer to these highly controlled growth methods represents a route to mastering interface quality in 2D ferroics.

### 4.3 Interface Engineering and Environmental Stability Strategies

Even with high‐quality materials and precise assembly techniques, an extrinsic environment can severely degrade multiferroic coupling. A major challenge is the pronounced air sensitivity of 2D ferromagnets. For example, Huang et al. highlighted the rapid degradation of monolayer CrI_3_ [77] and Fei et al. similarly implemented strict inert‐gas fabrication protocols to prevent the degradation of Fe_3_GeTe_2_ [193]. Therefore, robust passivation strategies are required. Encapsulation with hBN, as demonstrated by Mayorov et al. [194], remains effective but is difficult to scale up. Recently, atomic layer deposition (ALD) has emerged as an alternative method for scalability. Li et al. developed a seeding method to grow high‐quality Al_2_O_3_ directly on chemically inert 2D surfaces without damaging the underlying atomic structure, providing wafer‐scale passivation [195]. Furthermore, Deng et al. demonstrated that an ionic liquid gate can serve as an effective doping medium, dramatically enhancing the Curie temperature of Fe_3_GeTe_2_ to room temperature via electrostatic charge transfer [75].

Beyond environmental stability, optimizing the electrical contact interface is crucial for device efficiency. Allain et al. highlighted that minimizing the Schottky barrier at the metal‐2D interface is essential [196], and Cui et al. demonstrated low‐temperature ohmic contacts using indium electrodes [197]. Post‐fabrication treatments also can be used to optimize interface quality. Kretinin et al. showed that thermal annealing drives interfacial hydrocarbons into segregated bubbles, leaving large, atomically clean areas [198]. Li et al. demonstrated tuning exchange coupling via interlayer spacing controlled by hydrostatic pressure [199]. More recently, Wang et al. and Alghamdi et al. demonstrated that the interface between 2D magnets (e.g., Fe_3_GeTe_2_) and heavy metal electrodes can be engineered to enable efficient spin–orbit torque switching, a critical mechanism for writing in multiferroic memories [200, 201]. Taken together, these strategies are central to realize the stable 2D multiferroic heterostructures in realistic operating environments. In essence, the transition from fundamental exploration to practical device application hinges on our ability to integrate these fabrication pillars—synthesis, assembly, and interface engineering—into a scalable and reliable process flow. As we master the art of constructing pristine vdW interfaces, we can better access the intrinsic limits of 2D multiferroics, which can serve as next‐generation spintronics and memory technologies by leveraging the unique advantages of low‐dimensional quantum materials.

## 5 Conclusions and Outlook

Since the discovery of intrinsic 2D ferromagnets, a wide range of studies has shown that magnetic phases in the 2D limit can be efficiently tuned by electric fields, strain, pressure, and interfacial coupling. Beyond their fundamental importance as platforms for probing spin dynamics under strong quantum confinement, vdW magnets and their multiferroic heterostructures offer promising opportunities for low‐power spintronic functionalities. In particular, electric‐field control of magnetism in ferromagnet/ferroelectric vdW heterostructures provides a compelling route to reduce switching energy relative to current‐driven approaches. However, translating these materials‐level advances into reproducible and scalable device technologies requires several critical challenges to be addressed. To structure the outlook, we identify four key challenges and corresponding solution pathways: scalability, room‐temperature operation, quantitative and mechanistic characterization, and reproducibility.

First, scalability remains a primary bottleneck. Many vdW multiferroic demonstrations still rely on mechanically exfoliated flakes, which provide high crystalline quality but are limited in lateral size, throughput, and device‐to‐device uniformity. Practical spintronic applications will require wafer‐scale control over thickness, stoichiometry, phase purity, and interface structure. Phase‐engineered CVD and MOCVD growth, together with epitaxial routes such as wafer‐scale MBE‐grown Fe_3_GeTe_2_ [173], provide realistic pathways toward large‐area integration. These approaches are particularly important because they can stabilize metastable magnetic phases, control defect‐mediated ferroic order, and reduce the reliance on manual stacking. In the longer term, direct growth of ferromagnet/ferroelectric vdW heterostructures with atomically defined interfaces will be essential for combining scalability with strong and reproducible ME coupling.

Second, robust room‐temperature operation must be secured. Although prototypical 2D ferromagnets such as CrI_3_ and Cr_2_Ge_2_Te_6_ have played a foundational role in establishing 2D magnetism, their magnetic ordering temperatures are far below room temperature, limiting their direct relevance for practical devices. Future progress therefore depends on expanding the library of vdW magnets that remain stable at or above room temperature. Carrier‐density modulation by electrostatic or ionic gating has raised the Curie temperature of Fe_3_GeTe_2_ to room temperature [75], while strain engineering, pressure control, defect engineering, and proximity coupling provide additional routes for stabilizing magnetic order. Intrinsically high‐Curie‐temperature vdW magnets such as Fe_3_GaTe_2_ [202] further relax this constraint at the materials level. Combining such room‐temperature magnetic layers with robust vdW or CMOS‐compatible ferroelectrics will be crucial for moving beyond proof‐of‐concept demonstrations.

Third, quantitative benchmarking and mechanistic understanding remain insufficient. In oxide multiferroic heterostructures, ME coupling coefficients, coercive field modulation, switching energy, endurance, and retention are often reported as device‐relevant metrics. In contrast, many vdW studies still rely on qualitative signatures such as hysteresis loop shifts, anisotropy changes, domain evolution, or theoretically predicted changes in magnetic ordering. Establishing standardized protocols for evaluating ME coupling coefficient, switching energy, operating temperature, endurance, retention, switching speed, and cycle‐to‐cycle variation is therefore essential for meaningful comparison across material systems. Such benchmarking should also distinguish clearly between room‐temperature experimental demonstrations, cryogenic measurements, and theoretical predictions. Equally important is the direct identification of the microscopic coupling mechanism. Although polarization‐driven magnetic‐domain evolution has started to be explored [93, 94], comprehensive studies that directly correlate ferroelectric and magnetic domain configurations remain limited. Multimodal visualization of polarization domains, magnetic domains, domain‐wall motion, and interfacial charge redistribution will be critical for clarifying how ferroic domains govern switching dynamics and variability.

Fourth, reproducibility and environmental stability must be improved. Because ME coupling in vdW heterostructures is often mediated by short‐range interfacial effects, even small amounts of polymer residue, trapped adsorbates, interfacial disorder, or ambient degradation can screen polarization fields and distort magnetic responses [92]. This sensitivity makes device‐to‐device variation a central obstacle for vdW multiferroic spintronics. Standardized dry‐transfer protocols, contamination‐minimized assembly, in situ interface cleaning, and post‐transfer annealing can mitigate extrinsic disorder in mechanically assembled devices. At the same time, passivation and encapsulation strategies, including hBN encapsulation and scalable ALD‐based protection layers, are needed to preserve air‐sensitive vdW magnets and ferroelectrics under realistic operating conditions. Ultimately, direct‐growth strategies that eliminate transfer residues altogether may provide the most reliable route toward clean and scalable interfaces.

Addressing these challenges collectively, rather than in isolation, will define the roadmap for vdW multiferroic spintronics. Scalable growth must be combined with room‐temperature magnetic and ferroelectric order; quantitative benchmarking must be paired with microscopic mechanism identification; and clean‐interface fabrication must be integrated with environmental protection. Progress along these directions will determine whether vdW multiferroic heterostructures can evolve from scientifically intriguing platforms into deployable technologies for low‐power memory, logic, and spintronic devices.

## Funding

The National Research Foundation of Korea (NRF) grant funded by the Korea government (MSIT) (No. RS‐2025‐00563779), the InnoCORE program of the Ministry of Science and ICT (1.260007.01).

## Conflicts of Interest

The authors declare no conflicts of interest.

## Data Availability

The data that support the findings of this study are available from the corresponding author upon reasonable request.
